# 2,5-[C_4_+C_2_] Ringtransformation of Pyrylium Salts with α-Sulfinylacetaldehydes

**DOI:** 10.3390/molecules28227590

**Published:** 2023-11-14

**Authors:** Dominik Bauer, Kathrin Hofmann, Michael Reggelin

**Affiliations:** 1 Clemens-Schöpf-Institute for Organic Chemistry and Biochemistry, Technical University of Darmstadt, 64287 Darmstadt, Germany; db@chemie.tu-darmstadt.de; 2 Eduard-Zintl-Institute for Inorganic and Physical Chemistry, Technical University of Darmstadt, 64287 Darmstadt, Germany; kathrin.hofmann@tu-darmstadt.de

**Keywords:** α-sulfinylacetaldehyde, sulfoxide, pyrylium salt, ring transformation, *meta*-terphenyl

## Abstract

A rapid synthesis of chiral sulfoxide-functionalized *meta*-terphenyl derivatives by a 2,5-[C_4_+C_2_] ring transformation reaction of pyrylium salts with in situ generated enantiomerically pure α-sulfinylacetaldehydes is described in this paper. This synthetic method demonstrates, for the first time, the use of α-sulfinylacetaldehydes in a reaction sequence initiated by the nucleophilic attack of pyrylium salts by α-sulfinylcarbanions to generate chiral aromatic systems. The method presented shows a broad applicability starting with various methyl sulfoxides and a number of functionalized pyrylium salts, furnishing *meta*-terphenyls with complex substitution patterns from readily accessible starting compounds.

## 1. Introduction

**Figure 1:**
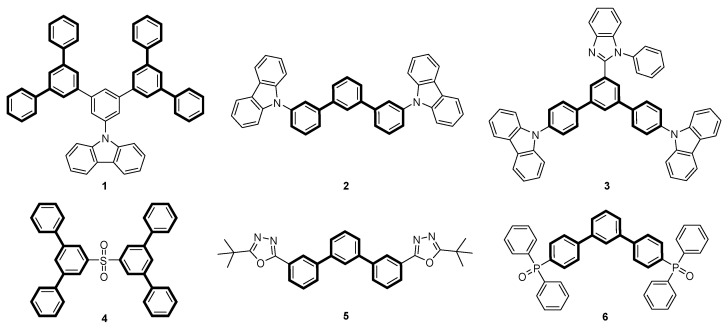
Examples of host **1**–**4** [10,11] and electron transport materials **5**–**6** [12] containing *meta*-terphenyl units.

Terphenyls are a class of organic compounds consisting of three interconnected phenyl rings. Depending on the arrangements of the aromatic rings, where substitutions can occur in the *ortho*-, *meta*-, or *para*-positions, different structural isomers and properties can be obtained. The *meta*-terphenyl skeleton occurs in several natural compounds [1], such as trifucol [2], macranthol [3], and mulberrofuran R [4]. Due to their extensive conjugation, terphenyls exhibit distinct optical [5,6,7,8] and electronic properties [9] that make them valuable for the preparation of various materials, such as organic light-emitting diodes (OLEDs, Figure 1).

**Scheme 1:**
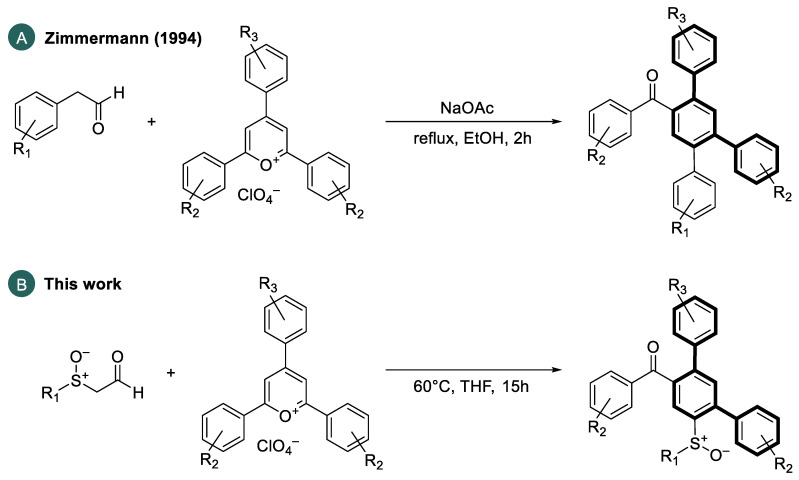
(**A**) Ring transformations of arylacetaldehydes with triarylpyrylium salts by Zimmermann [25]. (**B**) Use of α-sulfinylacetaldehydes to perform an analogous ring transformation reaction for the preparation of sulfinyl-functionalized *meta*-terphenyls.

In this case, the *meta*-terphenyl skeleton is mainly used in host materials. Especially, carbazole-substituted *meta*-terphenyl derivatives of types **1**–**3** show promising optoelectronic properties [11,13,14,15]. In addition, Sasabe et al. [10] synthesized a sulfone-bridged *meta*-terphenyl derivative **4** as a high-performance host material for green and blue OLEDs. In addition, *meta*-terphenyl derivatives are used in electron transport materials. Examples include *m*-terphenyloxadiazole **5**, synthesized by Wu et al. [5], or *m*-terphenyldiphenylphosphine oxide **6**, prepared by Zhang et al. [12]. An interesting extension of the above-mentioned applications would be the synthesis of chiral, non-racemic polyaromatic systems aimed at the synthesis of materials emitting circularly polarized light (CPL), which is of interest for many optoelectronic applications [16,17,18]. In general, two primary approaches to the preparation of terphenyl compounds can be distinguished: (i) the coupling of dihalobenzene derivatives with aryl metal nucleophiles [19,20,21,22] and (ii) the use of open-chain precursor molecules to form the aromatic rings by concerted or sequential benzannulation reactions [22,23,24]. In 1994, Zimmermann [25] reported a ring transformation reaction of triarylpyrylium salts with aryl acetaldehydes to obtain substituted carbocycles. He converted phenylacetaldehyde or 4-fluorophenylacetaldehyde with functionalized 2,4,6-triarylpyrylium salts in an ethanolic solution in the presence of sodium acetate as a weak base into the corresponding 2,4,5-triarylbenzophenones in high yields (Scheme 1).

Based on these results, in this paper, we use chiral, enantiomerically pure α-sulfinylacetaldehydes, as nucleophiles to perform an analogous ring transformation with a series of 2,4,6-triarylpyrylium salts to obtain optically active sulfinylated *meta*-terphenyls.

## 2. Results and Discussion

**Scheme 2:**
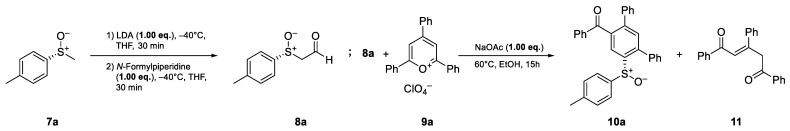
Initial experiments employing the isolated sulfinylacetaldehyde **8a**.

The preparation of enantiomerically pure (*R*)-*p*-tolyl methyl sulfoxide ((*R*)-**7a**) was conducted using the method developed by Andersen [26] and Solladie [27,28] starting from *p*-toluene sodium sulfinate. Subsequently, the synthesis of (*R*)-sulfinylacetaldehyde (**8a**) was achieved via the deprotonation of (*R*)-**7a** with lithiumdiisopropylamide (LDA), followed by a formyl transfer with *N*-formylpiperidine, according to the method of Pflieger et al. [29]. After the isolation of aldehyde **8a**, the ring transformation was performed with 2,4,6-triphenylpyrylium perchlorate (**9a**) [30] in ethanol in the presence of sodium acetate, analogous to the work of Zimmermann [25]. In this first attempt, the desired cyclization product **10a** was obtained at a 20% yield. In addition to the desired product, the open chain 1,4-diketone **11** was isolated at a 28% yield as a side product (Scheme 2, see also Scheme 5). 

Due to the instability of **8a** [31], we tried to avoid its isolation. After the reaction of lithiated sulfoxide (*R*)-**7a** with the formylating reagent, a THF solution at −40 °C was added directly to a room temperature suspension of pyrylium salt **9a** in THF and heated to 60 °C overnight. This significantly increased the yield of **10a** to 32%. Nevertheless, the by-product **11** was isolated at approximately the same yield as before (Table 1, #1). By adding 4  Å molecular sieves, its yield was reduced from 26% to 10% (Table 1, #2). Surprisingly, increasing the equivalents of (*R*)-sulfinylacetaldehyde lowered the yield of the cyclization product dramatically, whereas the yield of the by-product remained unchanged (Table 1, #3). One reason for the low yield seems to be the high concentration of sulfinylacetaldehyde, which is very unstable at elevated temperatures and may undergo self-condensation. By not isolating **8a**, it is no longer necessary to use sodium acetate as a base due to the formation of lithiumpiperidide in the course of the reaction. This leads to approximately the same product yield as entry 2, but the yield of the by-product decreases significantly (Table 1, #4). Conducting the reaction with a 1:1.8 excess of the pyrylium salt increased the yield of **10a** even further, to 49% (Table 1, #5).

**Figure 2:**
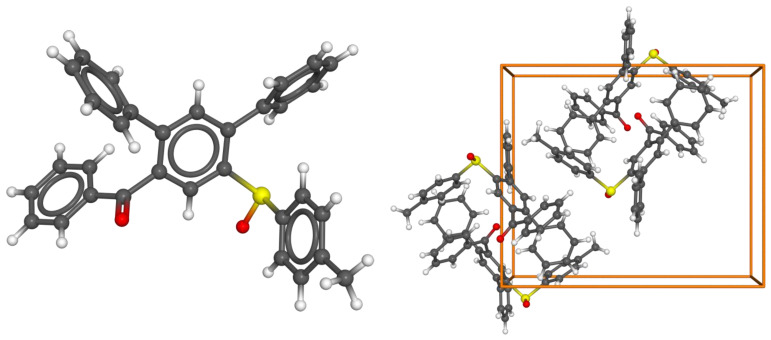
(**left**) Molecular structure of **10a**. (**right**) Unit cell (Z = 4, space group *P* 2_1_2_1_2_1_) with anisotropic thermal ellipsoids (50% probability level). The hydrogen atoms were placed geometrically.

The structure of the standard substrate **10a** was confirmed by a single-crystal X-ray analysis (Figure 2, CCDC deposition number 2302385).

**Scheme 3:**
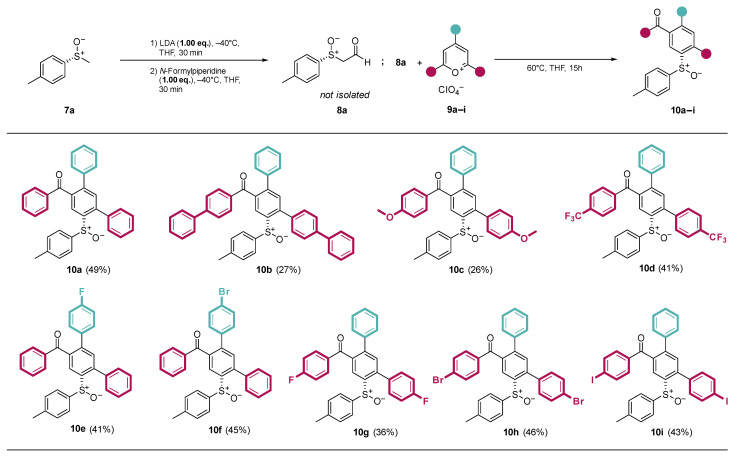
Substrate scope and isolated yields of the ring transformation products **10a**–**i** with different pyrylium salts **9a**–**i**.

At the optimized reaction conditions, substrate variations were first investigated using a variety of functionalized pyrylium salts **9a**–**i** (Scheme 3). With the exception of **10b** and **10c**, all products were obtained at yields ranging from 40 to 50%. The pyrylium salts reacting with **10b** and **10c** with electron-donating substituents (**9b**-Ph and **9c**-OMe) were expected to show a decreased reactivity due to the reduced electrophilicity at the 2-position of the pyrylium salt. Indeed, both donor-substituted products were obtained with reduced yields. 

**Scheme 4:**
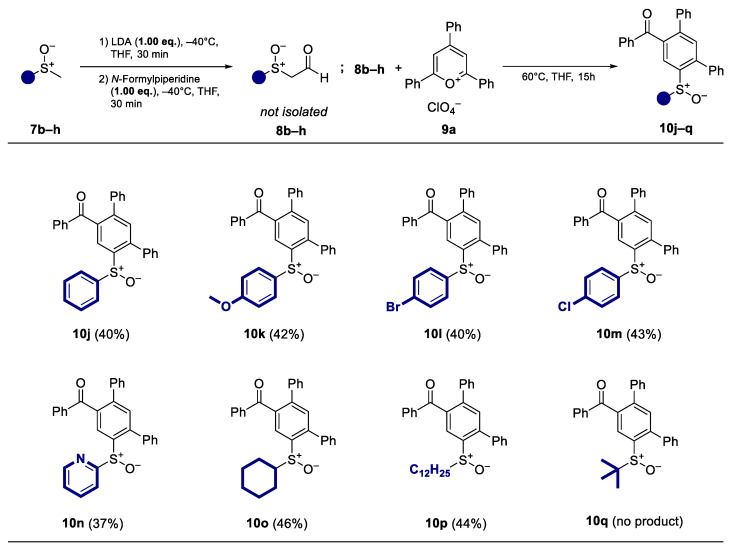
Substrate scope and isolated yields of the ring transformation products **10j**–**q** (racemic) with different methyl sulfoxides **7b**–**h**.

After varying the quantity of pyrylium salts, different racemic methyl sulfoxides **7b**–**h** were tested (Scheme 4). In this case, almost all sulfoxides showed comparable reactivity and the resulting terphenyls were obtained at yields similar to those of the standard substrate (*R*)-**7a**. The method is well suited aromatic sulfoxides with electron-donating substituents (**10k**) as well as with halogens (**10l**–**m**). Moreover, heteroaromatic sulfoxides (**10n**) and aliphatic sulfoxides with additional acidic protons in the α′-position showed comparable reactivity to (*R*)-**7a** and were obtained at yields of 40% (**10o**–**p**). No product can be isolated with sterically demanding *tert*-butyl methyl sulfoxide (**10q**).

**Scheme 5:**
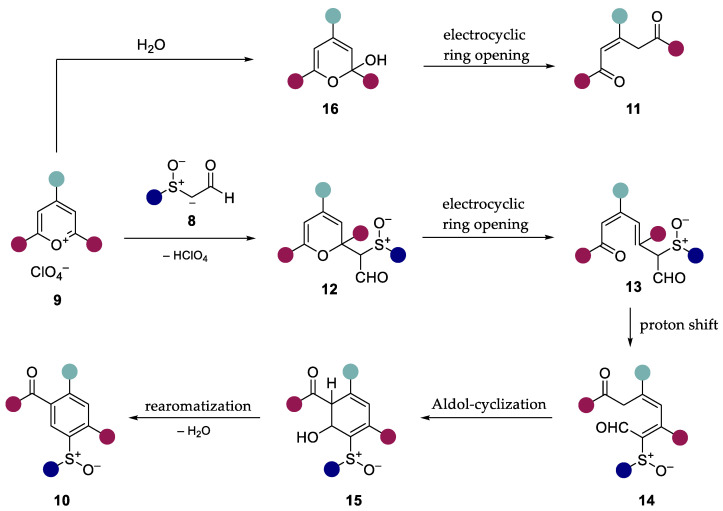
Assumed reaction mechanism of the ring transformation reaction of α-sulfinylacet-aldehydes with triarylpyrylium salts based on the work of Zimmermann [25].

From the results obtained, we assumed that the reaction mechanism described by Zimmermann [25] for phenylacetaldehyde could be applied to our system (Scheme 5). In this case, the carbanion of the α-sulfinylacetaldehydes **8** attacks the preferred 2-position [32] of the pyrylium salt **9**. The resulting *2H*-pyran **12** then reacts via electrocyclic ring opening to obtain the ketoaldehyde **13** [33]. The intermediate **14** obtained by proton shift reacts with the acidic methylene group in the course of an aldol addition to obtain the intermediate **15**. Condensation accompanied by rearomatization yields the ring transformation products **10**. In the presence of water, the pyrylium salts hydrolyze to the unstable cyclic hemiacetal **16**, which reacts by electrocyclic ring opening to form the open-chain 1,4-diketone **11** [34].

## 3. Materials and Methods

### 3.1. General Methods

The melting points were determined using a Stuart Smp10 melting point apparatus (Vernon Hills, IL, USA) and were uncorrected. Thin-layer chromatography (TLC) was performed using E. Merck silica gel SilG/UV254 by Macherey Nagel & Co., Düren, Germany (thickness of layer 0.2 mm) and visualized by UV fluorescence quenching. The ^1^H NMR spectra were recorded on a Bruker DRX 500 spectrometer operating at 500 MHz at 300 K. The ^13^C NMR spectra were recorded on the same instrument at 125 MHz. The ^19^F NMR spectra were recorded at 471 MHz. All chemical shifts (δ) are reported in ppm relative to tetramethylsilane (TMS) as the internal standard (δ = 0.00 ppm). The spectra were referenced against the residual solvent signal, as reported in the literature [35]. The fine structure of proton signals was specified as s (singlet), d (doublet), and t (triplet). Quaternary carbons were designated with subscript q. The iR spectra were recorded using a FTIR spectrometer Paragon 1000 (Perkin Elmer LAS GmbH, Rodgau, Germany). The specific optical rotations were determined using a Anton Paar MCP300 Polarimeter in 1 dm cuvettes. ESI-MS measurements were recorded using a Bruker Impact II. Elemental analyses were performed on Vario El from Elementar. The pyrylium salts **9a**–**i** were synthesized using the established methods [30] by the condensation of the corresponding benzaldehydes and acetophenones with phosphorus oxychloride and perchloric acid. The racemic methyl sulfoxides **7b**–**h** were synthesized from the corresponding thiols by methylation followed by oxidation with *m*CPBA, according to literature procedures [36]. All other reagents were obtained from commercial sources and used without further purification, unless otherwise specified. 

### 3.2. General Procedure for the Synthesis of the Ring Transformation Products

The corresponding methyl sulfoxide **7a**–**h** (1.00 eq.) was placed in an oven-dried Schlenk tube under argon and dissolved in dry THF (1.00 mL/mmol). The solution was cooled to −40 °C, and lithium diisopropylamide (1.00 eq.; 2M solution in THF, ethylbenzene, and *n*-heptane) was added and stirred for 30 min. *N*-formylpiperidine (1.00 eq.) was then added slowly, and the mixture was stirred for an additional 40 min at −40 °C. In a second Schlenk tube, the 2,4,6-triarylpyrylium salt **9a**–**i** was placed under argon and suspended with dry THF (2.00 mL/mmol). To this, the in situ synthesized α-sulfinylacetaldehyde was quickly added, and the mixture was heated to 60 °C. After 15 h, the mixture was cooled to room temperature, and dichloromethane (10 mL/mmol) was added and transferred to a separatory funnel. The mixture was extracted with water, and the aqueous phase was subsequently re-extracted twice with dichloromethane. The organic phases were combined and dried over magnesium sulfate. After the removal of the solvent, the crude product was purified by column chromatography (for aromatic methyl sulfoxides, 5% ethyl acetate to 20% ethyl acetate in *n*-pentane; for alkyl methyl sulfoxides, 25% Et_2_O in *n*-pentane).

*(R)-Phenyl(6′-(p-tolylsulfinyl)-[1,1′:3′,1″-terphenyl]-4′-yl)methanone* (**10a**) prepared from 600 mg (3.89 mmol) (*R*)-*p*-tolyl methyl sulfoxide ((*R*)-**7a**). Yield: 49% as an off-white solid. **mp** 186 °C. ***R_f_*** = 0.22 (20% ethyl acetate in *n*-pentane). **IR** (ATR) (cm^−1^): 1669, 1041, 697. **^1^H NMR** (500 MHz, Chloroform-*d*): δ 8.30 (s, 1H), 7.77–7.71 (m, 2H), 7.52–7.40 (m, 4H), 7.39–7.26 (m, 7H), 7.25–7.16 (m, 3H), 7.07–7.02 (m, 2H), 7.00–6.94 (m, 2H), 2.30 (s, 3H) ppm. **^13^C NMR** (126 MHz, Chloroform-*d*): δ 197.2_q_, 143.6_q_, 143.0_q_, 142.2_q_, 141.7_q_, 141.4_q_, 138.9_q_ (2C), 137.4_q_, 137.0_q_, 133.4, 132.4, 130.2, 129.7, 129.6, 129.0, 128.7, 128.7, 128.5, 128.5, 128.1, 125.7, 124.6, 21.5 ppm. **MS** (ESI) (*m*/*z*): 473.16 [M + H]^+^, 495.14 [M + Na]^+^, 511.11 [M + K]^+^, 945.31 [2M + H]^+^, 967.29 [2M + Na]^+^, 983.26 [2M + K]^+^. **HRMS** (ESI) (*m*/*z*): Calcd for C_32_H_24_O_2_S [M + H]^+^ 473.15698; Found 473.15715. **Anal. Calc**. for C_32_H_24_O_2_S: C 81.33; H 5.12. Found C 81.32; H 5.34. ${\left[\alpha \right]}_{D}^{20}$ = +28.27° (c 0.53; acetone).

*(R)-[1,1′-biphenyl]-4-yl(4′-(p-tolylsulfinyl)-[1,1′:3′,1″:4″,1″-quaterphenyl]-6′-yl)methanone* (**10b**) prepared from 600 mg (3.89 mmol) (*R*)-*p*-tolyl methyl sulfoxide ((*R*)-**7a**). Yield: 27% as an off-white solid. **mp** 116 °C. ***R_f_*** = 0.24 (20% ethyl acetate in *n*-pentane). **IR** (ATR) (cm^−1^): 1598, 754, 695. **^1^H NMR** (500 MHz, Chloroform-*d*): δ 8.34 (s, 1H), 7.85 (d, *J* = 8.5 Hz, 2H), 7.73–7.64 (m, 4H), 7.63–7.57 (m, 4H), 7.55–7.50 (m, 2H), 7.50–7.44 (m, 3H), 7.42 (d, *J* = 8.1 Hz, 4H), 7.34 (d, *J* = 6.6 Hz, 2H), 7.28–7.20 (m, 3H), 7.06 (s, 4H), 2.32 (s, 3H) ppm. **^13^C NMR** (126 MHz, Chloroform-*d*): δ 196.7_q_, 146.1_q_, 143.7_q_, 143.0_q_, 141.9_q_, 141.8_q_, 141.6_q_, 141.4_q_, 140.3_q_, 139.9_q_, 139.0_q_, 138.9_q_, 136.4_q_, 135.8_q_, 132.5, 130.8, 130.1, 129.8, 129.1, 129.0, 129.0, 128.6, 128.4, 128.1, 127.9, 127.4, 127.4, 127.2, 127.2, 125.7, 124.7, 21.5 ppm. **MS** (ESI) (*m*/*z*): 625.21 [M + H]^+^, 647.20 [M + Na]^+^, 1249.43 [2M + H]^+^, 1271.41 [2M + Na]^+^. **HRMS** (ESI) (*m*/*z*): Calcd for C_44_H_32_O_2_S [M + H]^+^ 625.21958; Found 625.21987. **Anal. Calc**. for C_44_H_32_O_2_S: C 84.58; H 5.16. Found C 84.28; H 5.27. ${\left[\alpha \right]}_{D}^{20}$ = −30.94° (c 0.48; acetone).

*(R)-(4-methoxy-6′-(p-tolylsulfinyl)-[1,1′:3′,1″-terphenyl]-4′-yl)(4-methoxyphenyl)methanone* (**10c**) prepared from 600 mg (3.89 mmol) (*R*)-*p*-tolyl methyl sulfoxide ((*R*)-**7a**). Yield: 26% as a yellow solid. **mp** 188 °C. ***R_f_*** = 0.06 (20% ethyl acetate in *n*-pentane). **IR** (ATR) (cm^−1^): 1594, 1248, 1169, 1026. **^1^H NMR** (500 MHz, Chloroform-*d*): δ 8.24 (s, 1H), 7.76 (d, *J* = 9.0 Hz, 2H), 7.37 (s, 1H), 7.32 (m, 2H), 7.30–7.21 (m, 5H), 7.09 (d, *J* = 8.3 Hz, 2H), 7.05 (d, *J* = 8.3 Hz, 2H), 6.99 (d, *J* = 8.8 Hz, 2H), 6.87 (d, *J* = 9.0 Hz, 2H), 3.92 (s, 3H), 3.87 (s, 3H), 2.33 (s, 3H) ppm. **^13^C NMR** (126 MHz, Chloroform-*d*): δ 195.8_q_, 163.8_q_, 160.0_q_, 143.4_q_, 142.8_q_, 141.7_q_, 141.6_q_, 141.5_q_, 139.1_q_, 139.0_q_, 132.6, 132.5, 130.9, 130.1_q_, 129.9_q_, 129.7, 128.9, 128.5, 128.0, 125.6, 124.5, 114.1, 113.8, 55.6, 55.5, 21.5 ppm. **MS** (ESI) (*m*/*z*): 533.17 [M + H]^+^, 555.16 [M + Na]^+^, 1065.35 [2M + H]^+^, 1087.33 [2M + Na]^+^. **HRMS** (ESI) (*m*/*z*): Calcd for C_34_H_28_O_4_S [M + H]^+^ 533.17811; Found 533.17854. **Anal. Calc**. for C_34_H_28_O_4_S: C 76.67; H 5.30. Found C 76.33; H 5.12. ${\left[\alpha \right]}_{D}^{20}$ = +15.19° (c 0.25; acetone).

*(R)-(6′-(p-tolylsulfinyl)-4-(trifluoromethyl)-[1,1′:3′,1″-terphenyl]-4′-yl)(4-(trifluoromethyl)phenyl)methanone* (**10d**) prepared from 300 mg (1.95 mmol) (*R*)-*p*-tolyl methyl sulfoxide ((*R*)-**7a**). Yield: 41% as an off-white solid. **mp** 91 °C. ***R_f_*** = 0.47 (20% ethyl acetate in *n*-pentane). **IR** (ATR) (cm^−1^): 1672, 1322, 1064. **^1^H NMR** (500 MHz, Chloroform-*d*): δ 8.34 (s, 1H), 7.78 (d, *J* = 8.1 Hz, 2H), 7.69 (d, *J* = 8.0 Hz, 2H), 7.58 (d, *J* = 8.1 Hz, 2H), 7.42 (d, *J* = 8.0 Hz, 2H), 7.38 (s, 1H), 7.23 (m, 5H), 7.07 (d, *J* = 8.0 Hz, 2H), 6.99 (d, *J* = 8.0 Hz, 2H), 2.32 (s, 3H) ppm. **^13^C NMR** (126 MHz, Chloroform-*d*): δ 196.0_q_, 143.9_q_, 143.4_q_, 142.3_q_, 141.2_q_, 140.9_q_, 140.7_q_, 139.6_q_, 138.7_q_, 138.3_q_, 134.5_q_ (d, ^2^*J*_CF_ = 33 Hz), 132.3, 131.0_q_ (d, ^2^*J*_CF_ = 33 Hz), 130.2, 130.0 (2C), 129.0, 128.8, 128.5, 125.9, 125.7 (q, ^3^*J*_CF_ = 3.9 Hz), 125.5 (q, ^3^*J*_CF_ = 3.9 Hz), 125.3, 124.0_q_ (^1^*J*_CF_ = 271 Hz), 123.5_q_ (^1^*J*_CF_ = 271 Hz), 21.5 ppm. ^19^**F NMR** (471 MHz, Chloroform-*d*) δ -62.6, -63.2 ppm. **MS** (ESI) (*m*/*z*): 609.13 [M + H]^+^, 631.11 [M + Na]^+^, 647.09 [M + K]^+^, 1217.26 [2M + H]^+^, 1239.24 [2M + Na]^+^, 1255.21 [2M + K]^+^. **HRMS** (ESI) (*m*/*z*): Calcd for C_34_H_22_F_6_O_2_S [M + H]^+^ 609.13175; Found 609.13177. **Anal. Calc.** for C_34_H_22_F_6_O_2_S: C 67.10; H 3.64. Found C 67.17; H 3.66. ${\left[\alpha \right]}_{D}^{20}$ = +8.70° (c 0.53; Aceton).

*(R)-(4″-fluoro-6′-(p-tolylsulfinyl)-[1,1′:3′,1″-terphenyl]-4′-yl)(phenyl)methanone* (**10e**) prepared from 600 mg (3.98 mmol) (*R*)-*p*-tolyl methyl sulfoxide ((*R*)-**7a**). Yield: 41% as an off-white solid. **mp** 164 °C. ***R_f_*** = 0.20 (20% ethyl acetate in *n*-pentane). **IR** (ATR) (cm^−1^): 1667, 1039. **^1^H NMR** (500 MHz, Chloroform-*d*): δ 8.29 (s, 1H), 7.77–7.69 (m, 2H), 7.56–7.48 (m, 1H), 7.46–7.41 (m, 3H), 7.40–7.34 (m, 2H), 7.33 (s, 1H), 7.32–7.28 (m, 2H), 7.27–7.22 (m, 2H), 7.04 (d, *J* = 8.0 Hz, 2H), 7.00–6.94 (m, 2H), 6.94–6.88 (m, 2H), 2.30 (s, 3H) ppm. **^13^C NMR** (126 MHz, Chloroform-*d*): δ 197.1_q_, 162.6_q_ (d, ^1^*J*_CF_ = 248.0 Hz), 143.1_q_, 142.5_q_, 142.3_q_, 141.8_q_, 141.3_q_, 138.8_q_, 137.3_q_, 136.9_q_, 135.0_q_ (d, ^4^*J*_CF_ = 3.3 Hz), 133.6, 132.4, 130.7 (^3^*J*_CF_ = 8.2 Hz), 130.2, 129.8, 129.6, 128.8, 128.6, 128.5, 126.9, 125.7, 124.6, 115.6 (d, ^2^*J*_CF_ = 21.6 Hz), 21.5 ppm. ^19^**F NMR** (471 MHz, Chloroform-*d*) δ -113.8 ppm. **MS** (ESI) (*m*/*z*): 491.15 [M + H]^+^, 513.13 [M + Na]^+^, 529.10 [M + K]^+^, 981.29 [2M + H]^+^, 1003.28 [2M + Na]^+^, 1019.24 [2M + K]^+^. **HRMS** (ESI) (*m*/*z*): Calcd for C_32_H_23_FO_2_S [M + H]^+^ 491.14756; Found 491.14762. **Anal. Calc.** for C_32_H_23_FO_2_S: C 78.34; H 4.73. Found C 78.46; H 4.96. ${\left[\alpha \right]}_{D}^{20}$ = +29.52° (c 0.49; acetone).

*(R)-(4″-bromo-6′-(p-tolylsulfinyl)-[1,1′:3′,1″-terphenyl]-4′-yl)(phenyl)methanone* (**10f**) prepared from 600 mg (3.89 mmol) (*R*)-*p*-tolyl methyl sulfoxide ((*R*)-**7a**). Yield: 46% as a white solid. **mp** 167 °C. ***R_f_*** = 0.21 (20% ethyl acetate in *n*-pentane). **IR** (ATR) (cm^−1^): 1660, 1046. **^1^H NMR** (500 MHz, Chloroform-*d*): δ 8.29 (s, 1H), 7.77–7.72 (m, 2H), 7.59–7.50 (m, 1H), 7.49–7.34 (m, 7H), 7.32 (s, 1H), 7.31–7.27 (m, 2H), 7.19–7.11 (m, 2H), 7.04 (d, *J* = 8.0 Hz, 2H), 6.99–6.89 (m, 2H), 2.30 (s, 3H) ppm. **^13^C NMR** (126 MHz, Chloroform-*d*): δ 196.9_q_, 143.4_q_, 142.4_q_, 142.3_q_, 141.8_q_, 141.2_q_, 138.7_q_, 137.9_q_, 137.2_q_, 136.9_q_, 133.7, 132.3, 131.7, 130.5, 130.2, 129.8, 129.6, 128.8, 128.8, 128.6, 125.6, 124.6, 122.6_q_, 21.5 ppm. **MS** (ESI) (*m*/*z*): 551.07 [M + H]^+^, 573.05 [M + Na]^+^, 1101.13 [2M + H]^+^, 1123.11 [2M + Na]^+^. **HRMS** (ESI) (*m*/*z*): Calcd for C_32_H_23_BrO_2_S [M + H]^+^ 551.06749; Found 551.06752. **Anal. Calc**. for C_32_H_23_BrO_2_S: C 69.69; H 4.20. Found C 69.21; H 3.99. ${\left[\alpha \right]}_{D}^{20}$ = +25.77° (c 0.50; acetone).

*(R)-(4-fluoro-6′-(p-tolylsulfinyl)-[1,1′:3′,1″-terphenyl]-4′-yl)(4-fluorophenyl)methanone* (**10g**) prepared from 600 mg (3.89 mmol) (*R*)-*p*-tolyl methyl sulfoxide ((*R*)-**7a**). Yield: 36% as an off-white solid. **mp** 190 °C. ***R_f_*** = 0.26 (20% ethyl acetate in *n*-pentane). **IR** (ATR) (cm^−1^): 1663, 1594, 1226. **^1^H NMR** (500 MHz, Chloroform-*d*): δ 8.26 (s, 1H), 7.77–7.64 (m, 2H), 7.29–7.17 (m, 7H), 7.10 (t, *J* = 8.6 Hz, 2H), 7.06 (d, *J* = 8.0 Hz, 2H), 6.99 (t, *J* = 8.6 Hz, 4H), 2.30 (s, 3H) ppm. **^13^C NMR** (126 MHz, Chloroform-*d*): δ 195.6_q_, 165.9_q_ (d, ^1^*J*_CF_ = 256.1 Hz), 163.12_q_ (d, ^1^*J*_CF_ = 248.9 Hz), 143.59_q_, 143.20_q_, 142.11_q_, 141.33_q_, 141.11_q_, 138.83_q_, 138.69_q_, 133.5_q_ (d, ^4^*J*_CF_ = 5.46 Hz), 133.4_q_ (d, ^4^*J*_CF_ = 5.95 Hz), 132.7 (d, ^3^*J*_CF_ = 9.5 Hz), 132.52, 131.4 (d, ^3^*J*_CF_ = 8.1 Hz), 129.92, 128.96, 128.68, 128.33, 125.82, 124.77, 115.8 (d, ^2^*J*_CF_ = 22.15 Hz), 115.7 (d, ^2^*J*_CF_ = 21.91 Hz), 21.56 ppm. ^19^**F NMR** (471 MHz, Chloroform-*d*) δ -104.2, -112.5 ppm. **MS** (ESI) (*m*/*z*): 509.14 [M + H]^+^, 531.12 [M + Na]^+^, 1017.27 [2M + H]^+^, 1039.25 [2M + Na]^+^. **HRMS** (ESI) (*m*/*z*): Calcd for C_32_H_22_F_2_O_2_S [M + H]^+^ 509.13813; Found 509.13831. **Anal. Calc**. for C_32_H_22_F_2_O_2_S: C 75.57; H 4.36. Found C 75.66; H 4.38. ${\left[\alpha \right]}_{D}^{20}$ = +28.14° (c 0.50; acetone).

*(R)-(4-bromo-6′-(p-tolylsulfinyl)-[1,1′:3′,1″-terphenyl]-4′-yl)(4-bromophenyl)methanone* (**10h**) prepared from 600 mg (3.89 mmol) (*R*)-*p*-tolyl methyl sulfoxide ((*R*)-**7a**). Yield: 46% as an off-white solid. **mp** 235 °C. ***R_f_*** = 0.41 (20% ethyl acetate in *n*-pentane). **IR** (ATR) (cm^−1^): 1667, 1039. **^1^H NMR** (500 MHz, Chloroform-*d*): δ 8.26 (s, 1H), 7.60–7.51 (m, 4H), 7.47 (d, *J* = 8.5 Hz, 2H), 7.33 (s, 1H), 7.23 (s, 5H), 7.18 (d, *J* = 8.5 Hz, 2H), 7.10 (d, *J* = 8.3 Hz, 2H), 7.03 (d, *J* = 8.3 Hz, 2H), 2.33 (s, 3H) ppm. **^13^C NMR** (126 MHz, Chloroform-*d*): 196.1_q_, 143.7_q_, 143.1_q_, 142.2_q_, 141.2_q_, 141.0_q_, 138.7_q_, 138.5_q_, 136.2_q_, 135.7_q_, 132.3, 131.9, 131.8, 131.5, 131.2, 129.9, 128.9, 128.8_q_, 128.7, 128.4, 125.7, 124.9, 123.2_q_, 21.5 ppm. **MS** (ESI) (*m*/*z*): 628.98 [M + H]^+^, 650.96 [M + Na]^+^, 666.93 [M + K]^+^, 1256.95 [2M + H]^+^, 1278.93 [2M + Na]^+^. **HRMS** (ESI) (*m*/*z*): Calcd for C_32_H_22_Br_2_O_2_S [M + H]^+^ 628.97800; Found 628.97792. **Anal. Calc**. for C_32_H_22_Br_2_O_2_S: C 60.97; H 3.52. Found C 60.89; H 3.66. ${\left[\alpha \right]}_{D}^{20}$ = −19.41° (c 0.50; acetone).

*(R)-(4-iodo-6′-(p-tolylsulfinyl)-[1,1′:3′,1″-terphenyl]-4′-yl)(4-iodophenyl)methanone* (**10i**) prepared from 300 mg (1.95 mmol) (*R*)-*p*-tolyl methyl sulfoxide ((*R*)-**7a**). Yield: 43% as an off-white solid. **mp** 234 °C. ***R_f_*** = 0.36 (20% ethyl acetate in *n*-pentane). **IR** (ATR) (cm^−1^): 1659, 1038, 956. **^1^H NMR** (500 MHz, Chloroform-*d*): δ 8.25 (s, 1H), 7.81–7.75 (m, 2H), 7.73–7.66 (m, 2H), 7.43–7.36 (m, 2H), 7.33 (s, 1H), 7.23 (m, 5H), 7.09 (d, *J* = 8.0 Hz, 2H), 7.07–7.00 (m, 4H), 2.33 (s, 3H) ppm. **^13^C NMR** (126 MHz, Chloroform-*d*): δ 196.2_q_, 143.6_q_, 142.9_q_, 142.0_q_, 141.2_q_, 140.9_q_, 138.5_q_, 138.4_q_, 137.8, 137.7, 136.7, 136.1, 132.1, 131.2, 131.1, 129.8, 128.8, 128.6, 128.3, 125.6, 124.8, 101.7_q_, 94.7_q_, 21.4 ppm. **MS** (ESI) (*m*/*z*): 724.95 [M + H]^+^, 746.93 [M + Na]^+^. **HRMS** (ESI) (*m*/*z*): Calcd for C_32_H_22_I_2_O_2_S [M + H]^+^ 724.95027; Found 724.95074. **Anal. Calc**. for C_32_H_22_I_2_O_2_S: C 53.06; H 3.06. Found C 53.04; H 2.99. ${\left[\alpha \right]}_{D}^{20}$ = −32.45° (c 0.26; acetone).

*Phenyl(6′-(phenylsulfinyl)-[1,1′:3′,1″-terphenyl]-4′-yl)methanone* (**10j**) prepared from 309 mg (2.20 mmol) methylphenylsulfoxide. Yield: 39% as an off-white solid. **mp** 140 °C. ***R_f_*** = 0.19 (20% ethyl acetate in *n*-pentane). **IR** (ATR) (cm^−1^): 1663, 1046. **^1^H NMR** (500 MHz, Chloroform-*d*): δ 8.31 (s, 1H), 7.79–7.72 (m, 2H), 7.56–7.18 (m, 17H), 7.17–7.04 (m, 2H) ppm. **^13^C NMR** (126 MHz, Chloroform-*d*): δ 197.2_q_, 144.5_q_, 143.8_q_, 142.7_q_, 142.3_q_, 139.0_q_, 138.9_q_, 137.4_q_, 137.0_q_, 133.4, 132.5, 131.2, 130.2, 129.6, 129.1, 129.0, 128.8, 128.8, 128.6, 128.5, 128.1, 125.6, 124.7 ppm. **MS** (ESI) (*m*/*z*): 459.14 [M + H]^+^. **HRMS** (ESI) (*m*/*z*): Calcd for C_31_H_22_O_2_S [M + H]^+^ 459.1413; Found 459.1414. 

*(6′-((4-methoxyphenyl)sulfinyl)-[1,1′:3′,1″-terphenyl]-4′-yl)(phenyl)methanone* (**10k**) prepared from 355 mg (2.09 mmol) 4-methoxyphenylmethylsulfoxide. Yield: 42% as an off-white solid. **mp** 94 °C. ***R_f_*** = 0.10 (20% ethyl acetate in *n*-pentane). **IR** (ATR) (cm^−1^): 1664, 1250, 1045, 698. **^1^H NMR** (500 MHz, Chloroform-*d*): δ 8.35 (s, 1H), 7.77 (d, *J* = 8.3 Hz, 2H), 7.57–7.48 (m, 1H), 7.45–7.37 (m, 6H), 7.33–7.27 (m, 4H), 7.27–7.16 (m, 3H), 7.02 (d, *J* = 8.8 Hz, 2H), 6.75 (d, *J* = 8.9 Hz, 2H), 3.79 (s, 3H) ppm. **^13^C NMR** (126 MHz, Chloroform-*d*): δ 197.2_q_, 161.8_q_, 143.4_q_, 142.9_q_, 142.0_q_, 138.8_q_, 138.7_q_, 137.3_q_, 136.9_q_, 135.5_q_, 133.3, 132.3, 130.1, 129.4, 128.9, 128.6, 128.5, 128.4, 128.4, 127.9, 127.7, 124.3, 114.4, 55.4 ppm. **MS** (ESI) (*m*/*z*): 489.15 [M + H]^+^, 511.13 [M + Na]^+^, 527.11 [M + K]^+^, 977.30 [2M + H]^+^, 999.28 [2M + Na]^+^, 1015.25 [2M + K]^+^. **HRMS** (ESI) (*m*/*z*): Calcd for C_32_H_24_O_3_S [M + H]^+^ 489.15189; Found 489.15195. **Anal. Calc**. for C_32_H_24_O_3_S: C 78.66; H 4.95. Found C 78.53; H 5.01.

*(6′-((4-bromophenyl)sulfinyl)-[1,1′:3′,1″-terphenyl]-4′-yl)(phenyl)methanone* (**10l**) prepared from 438 mg (2.00 mmol) 4-bromophenylmethylsulfoxid. Yield: 43% as a white solid. **mp** 92 °C. ***R_f_*** = 0.34 (20% ethyl acetate in *n*-pentane). **IR** (ATR) (cm^−1^): 1664, 1048, 697. **^1^H NMR** (500 MHz, Chloroform-*d*): δ 8.27 (s, 1H), 7.76–7.68 (m, 2H), 7.53–7.43 (m, 4H), 7.39 (s, 1H), 7.38–7.26 (m, 6H), 7.25–7.18 (m, 5H), 7.03–6.95 (m, 2H) ppm. **^13^C NMR** (126 MHz, Chloroform-*d*): δ 197.1_q_, 144.0_q_, 143.0_q_, 142.4_q_, 142.1_q_, 139.1_q_, 138.7_q_, 137.5_q_, 137.2_q_, 136.9_q_, 133.5, 132.6, 130.1, 129.6, 129.3, 129.0, 128.9, 128.6, 128.5, 128.2, 126.9, 124.5 ppm. **MS** (ESI) (*m*/*z*): 537.05 [M + H]^+^, 559.03 [M + Na]^+^, 575.00 [M + K]^+^, 1073.09 [2M + H]^+^, 1095.07 [2M + Na]^+^. **HRMS** (ESI) (*m*/*z*): Calcd for C_31_H_21_BrO_2_S [M + H]^+^ 537.05184; Found 537.05130. **Anal. Calc**. for C_31_H_21_BrO_2_S: C 75.52; H 4.29. Found C 75.48; H 4.32.

*(6′-((4-chlorophenyl)sulfinyl)-[1,1′:3′,1″-terphenyl]-4′-yl)(phenyl)methanone* (**10m**) prepared from 350 mg (2.00 mmol) 4-chlorophenylmethylsulfoxid. Yield: 40 % as a white solid. **mp** 122 °C. ***R_f_*** = 0.34 (20% ethyl acetate in *n*-pentane). **IR** (ATR) (cm^−1^): 1664, 1049, 697. **^1^H NMR** (500 MHz, Chloroform-*d*): δ 8.26 (s, 1H), 7.72 (d, *J* = 8.0 Hz, 2H), 7.53–7.43 (m, 4H), 7.41–7.30 (m, 7H), 7.28 (m, 2H), 7.25–7.19 (m, 3H), 6.92 (d, *J* = 8.0 Hz, 2H) ppm. **^13^C NMR** (126 MHz, Chloroform-*d*): δ 197.1_q_, 144.0_q_, 143.7_q_, 142.3_q_, 142.1_q_, 139.1_q_, 138.7_q_, 137.2_q_, 136.9_q_, 133.5, 132.6, 132.3, 130.1, 129.6, 129.0, 128.9, 128.6, 128.5, 128.2, 127.0, 125.8_q_, 124.5 ppm. **MS** (ESI) (*m*/*z*): 493.10 [M + H]^+^, 515.08 [M + Na]+, 531.05 [M + K]^+^, 985.19 [2M + H]^+^, 1007.17 [2M + Na]^+^. **HRMS** (ESI) (*m*/*z*): Calcd for C_31_H_21_ClO_2_S [M + H]^+^ 493.10236; Found 493.10232. **Anal. Calc**. for C_31_H_21_ClO_2_S: C 69.28; H 3.94. Found C 69.42; H 3.80.

*Phenyl(6′-(pyridin-2-ylsulfinyl)-[1,1′:3′,1″-terphenyl]-4′-yl)methanone* (**10n**) prepared from 300 mg (2.12 mmol) 2-pyridylmethylsulfoxid. Yield: 36% as a white solid. **mp** 110 °C. ***R_f_*** = 0.04 (20% ethyl acetate in *n*-pentane). **IR** (ATR) (cm^−1^): 1659, 1048, 689. **^1^H NMR** (500 MHz, Chloroform-*d*): δ 8.44 (m, 1H), 7.80 (s, 1H), 7.78–7.69 (m, 2H), 7.65–7.58 (m, 2H), 7.56–7.52 (m, 2H), 7.42 (s, 1H), 7.41–7.32 (m, 4H), 7.24–7.14 (m, 5H), 7.14–7.06 (m, 3H) ppm. **^13^C NMR** (126 MHz, Chloroform-*d*): δ 196.9_q_, 165.5_q_, 150.0, 144.4_q_, 144.2_q_, 142.1_q_, 138.9_q_ (2C), 138.1, 137.6_q_, 136.9_q_, 133.3, 132.5, 130.5, 130.1, 129.0, 128.7, 128.5, 128.4, 128.4, 128.2, 127.3, 124.7, 120.2 ppm. **MS** (ESI) (*m*/*z*): 460.13 [M + H]^+^. **HRMS** (ESI) (*m*/*z*): Calcd for C_30_H_21_NO_2_S [M + H]^+^ 460.1366; Found 460.1368. **Anal. Calc**. for C_30_H_21_NO_2_S: C 78.41; H 4.61; N 3.05. Found C 78.35; H 4.67; N 3.07.

*(6′-(cyclohexylsulfinyl)-[1,1′:3′,1″-terphenyl]-4′-yl)(phenyl)methanone* (**10o**) prepared from 304 mg (2.08 mmol) cyclohexylmethylsulfoxid. Yield: 46% as a white solid. **mp** 160 °C. ***R_f_*** = 0.22 (20% ethyl acetate in *n*-pentane). **IR** (ATR) (cm^−1^): 2926, 1664, 1046, 697. **^1^H NMR** (500 MHz, Chloroform-*d*): δ 8.11 (s, 1H), 7.77–7.69 (m, 2H), 7.52–7.42 (m, 7H), 7.38–7.32 (m, 4H), 7.28–7.20 (m, 3H), 2.27 (m, 1H), 1.84–1.73 (m, 1H), 1.70–1.61 (m, 1H), 1.60–1.50 (m, 2H), 1.48–1.16 (m, 3H), 1.16–0.99 (m, 3H), 0.92–0.81 (m, 1H) ppm. **^13^C NMR** (126 MHz, Chloroform-*d*): δ 197.3_q_, 143.6_q_, 142.1_q_, 139.3_q_, 138.9_q_, 138.6_q_, 137.6_q_, 137.0_q_, 133.4, 132.3, 130.1, 129.3, 129.0, 128.9, 128.7, 128.6, 128.4, 128.1, 126.0, 60.7, 27.3, 25.8, 25.3, 25.3, 22.8 ppm. **MS** (ESI) (*m*/*z*): 465.18 [M + H]^+^, 487.17 [M + Na]^+^, 503.14 [M + K]^+^, 951.35 [2M + Na]^+^. **HRMS** (ESI) (*m*/*z*): Calcd for C_31_H_28_O_2_S 465.18828; Found 465.18810. **Anal. Calc**. for C_31_H_28_O_2_S: C 80.14; H 6.07. Found C 80.22; H 6.02. 

*(6′-(dodecylsulfinyl)-[1,1′:3′,1″-terphenyl]-4′-yl)(phenyl)methanone* (**10p**) prepared from 465 mg (2.00 mmol) 1-dodecylmethylsulfoxid. Yield: 44% as a yellow oil. ***R_f_*** = 0.40 (20% ethyl acetate in *n*-pentane). **IR** (ATR) (cm^−1^): 2922, 1666, 1047, 697. **^1^H NMR** (500 MHz, Chloroform-*d*): δ 8.23 (s, 1H), 7.78 (d, *J* = 8.5 Hz, 2H), 7.58–7.45 (m, 6H), 7.45–7.33 (m, 4H), 7.28 (dd, *J* = 14.7, 7.7 Hz, 3H), 2.59 (dd, *J* = 14.0, 8.0 Hz, 1H), 2.56–2.39 (m, 1H), 1.72–1.58 (m, 1H), 1.54–1.41 (m, 1H), 1.39–1.07 (m, 18H), 0.93 (t, *J* = 7.0 Hz, 3H) ppm. **^13^C NMR** (126 MHz, Chloroform-*d*): δ 197.3_q_, 143.6_q_, 141.3_q_, 141.3_q_, 139.0_q_, 138.9_q_, 137.3_q_, 137.0_q_, 133.4, 132.3, 130.1, 129.1, 129.1, 129.0, 128.9, 128.6, 128.5, 128.4, 128.1, 125.0, 54.9, 32.0, 29.7 (2C), 29.6, 29.4, 29.4, 29.0, 28.3, 22.8, 22.2, 14.2 ppm. **MS** (ESI) (*m*/*z*): 551.30 [M + H]^+^, 573.28 [M + Na]^+^, 1001.59 [2M + H]^+^, 1123.57 [2M + Na]^+^. **HRMS** (ESI) (*m*/*z*): Calcd for C_37_H_43_O_2_S [M + H]^+^ 551.29783; Found 551.29773. **Anal. Calc**. for C_37_H_43_O_2_S: C 80.68; H 7.69. Found C 80.38; H 7.75.

## 4. Conclusions

In summary, we developed an efficient synthesis for the preparation of highly substituted *meta*-terphenyls bearing both a sulfoxide moiety and an acyl group at the central ring starting from readily available methyl sulfoxides **7a**–**h** and triarylpyrylium perchlorates **9a**–**i**. Starting from chiral, enantiomerically pure (*R*)-*p*-tolyl methyl sulfoxide ((*R*)-**7a**), the optically active derivatives **10a**–**i** were prepared. The method is broadly applicable to a variety of aromatic and alkyl methyl sulfoxides and differently functionalized pyrylium salts. All new compounds were fully characterized by spectroscopic methods. The crystal structural analysis of **10a** completed the structural evidence (see Supplementary Materials).

## Figures and Tables

**Table 1:** Optimization of reaction conditions ^a^.

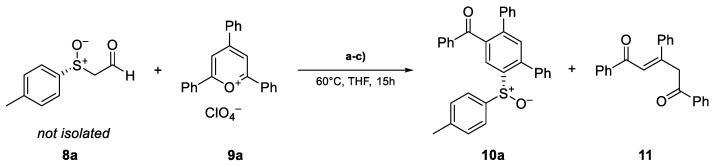					
Entry	eq. 8a	eq. 9a	Base	Yield 10a/%	Yield 11/%
1	1.0	1.0	NaOAc	32	26
2 ^b^	1.0	1.0	NaOAc	35	16
3	2.0	1.0	NaOAc	9	27
4	1.0	1.0	-	38	<10
**5**	**1.0**	**1.8**	**-**	**49**	**<10**

^a^ (a) 1.0 eq. LDA, −40 °C, 30 min. (b) 1.0 eq. *N*-formylpiperidine, −40 °C, 40 min. (c) 1.0–1.8 eq. **9a**, 0.0–1.0 eq. NaOAc (suspension in THF), 15 h, 60 °C. ^b^ 4 Å molecular sieves were added.

## Data Availability

The data presented in this study are available upon request from the corresponding author.

## Supplementary Materials

The following supporting information can be downloaded at: https://www.mdpi.com/article/10.3390/molecules28227590/s1. NMR spectra and crystallographic data.
