# Supplementary material for: 2,5-[C4+C2] Ringtransformation of Pyrylium Salts with α-Sulfinylacetaldehydes
Source: Molecules. 2023 Nov 14;28(22):7590. doi: 10.3390/molecules28227590 (PMC10673159; doi:10.3390/molecules28227590)
Supplement: Supplementary file 1 [file molecules-28-07590-s001.zip › molecules-2711326-supplementary.pdf]

Supporting information

2,5-[C_4+C_2] Ringtransformation of Pyrylium Salts with α-Sulfinylacetaldehydes

Dominik Bauer [1], Kathrin Hofmann [2] and Michael Reggelin [1,*]

[1] Clemens-Schöpf-Institute for Organic Chemistry and Biochemistry, Technical University of Darmstadt, 64287 Darmstadt, Germany; db@chemie.tu-darmstadt.de
[2] Eduard-Zintl-Institute for Inorganic and Physical Chemistry, Technical University of Darmstadt, 64287 Darmstadt, Germany; kathrin.hofmann@tu-darmstadt.de
* Correspondence: re@chemie.tu-darmstadt.de

Table of contents

NMR spectra of compounds **10a-p**

Crystallographic data of compound **10a**

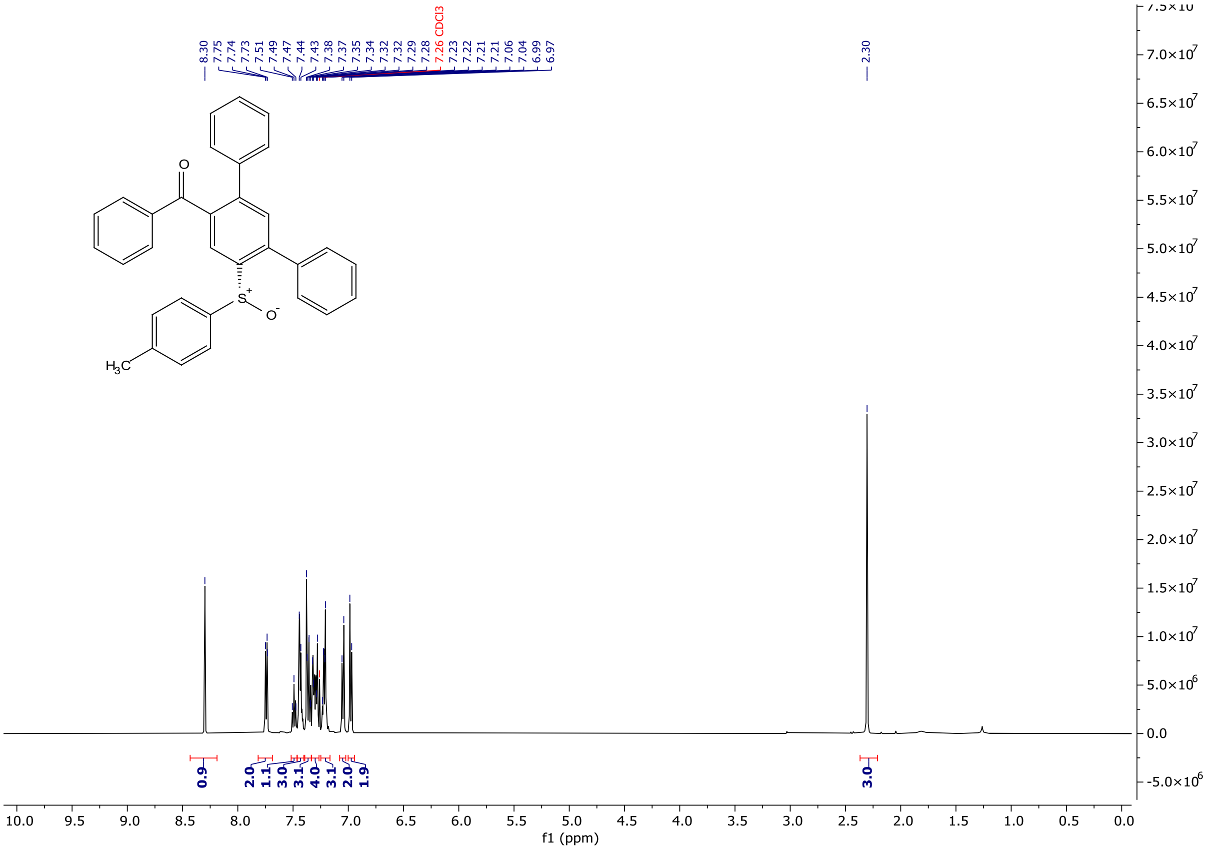

Figure S1. ^{1}H NMR (500 MHz, Chloroform-*d*) of compound **10a**.

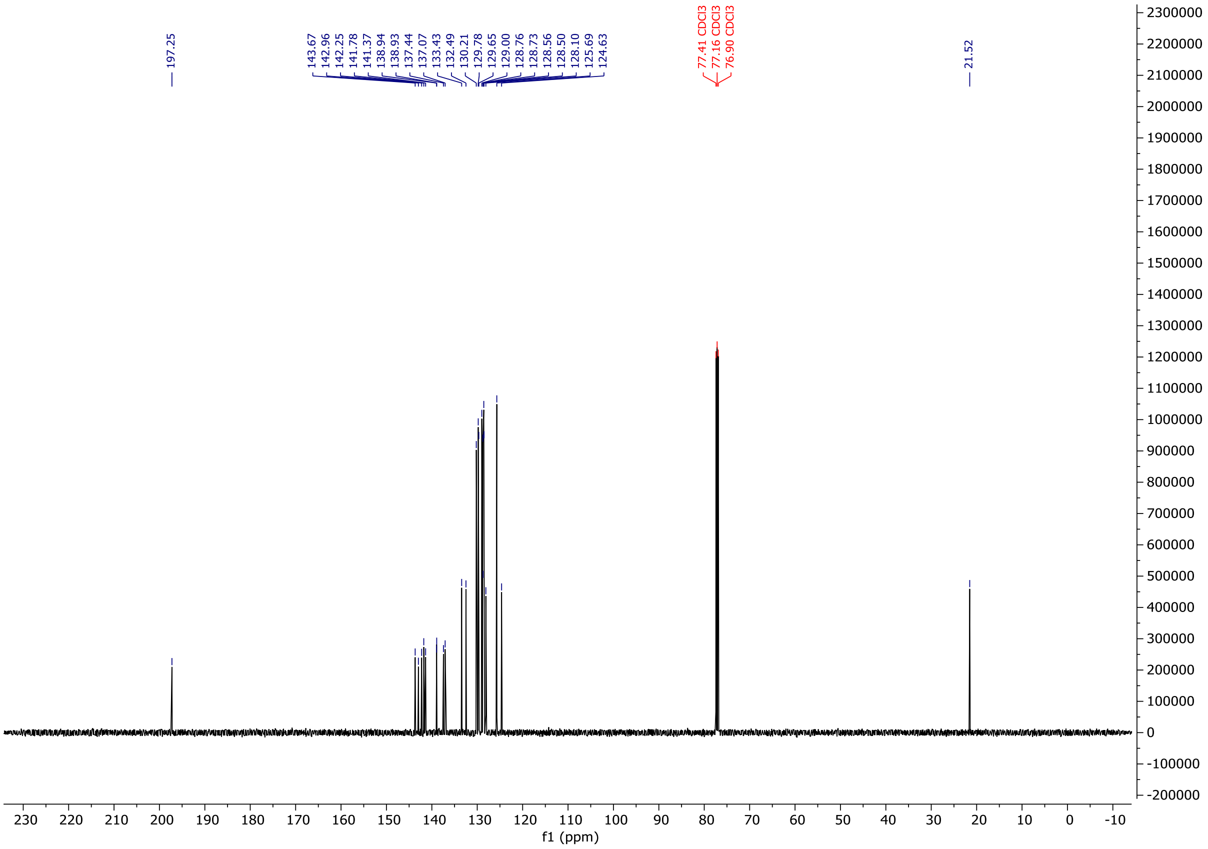

Figure S2. ^{13}C NMR (126 MHz, Chloroform-*d*) of compound **10a**.

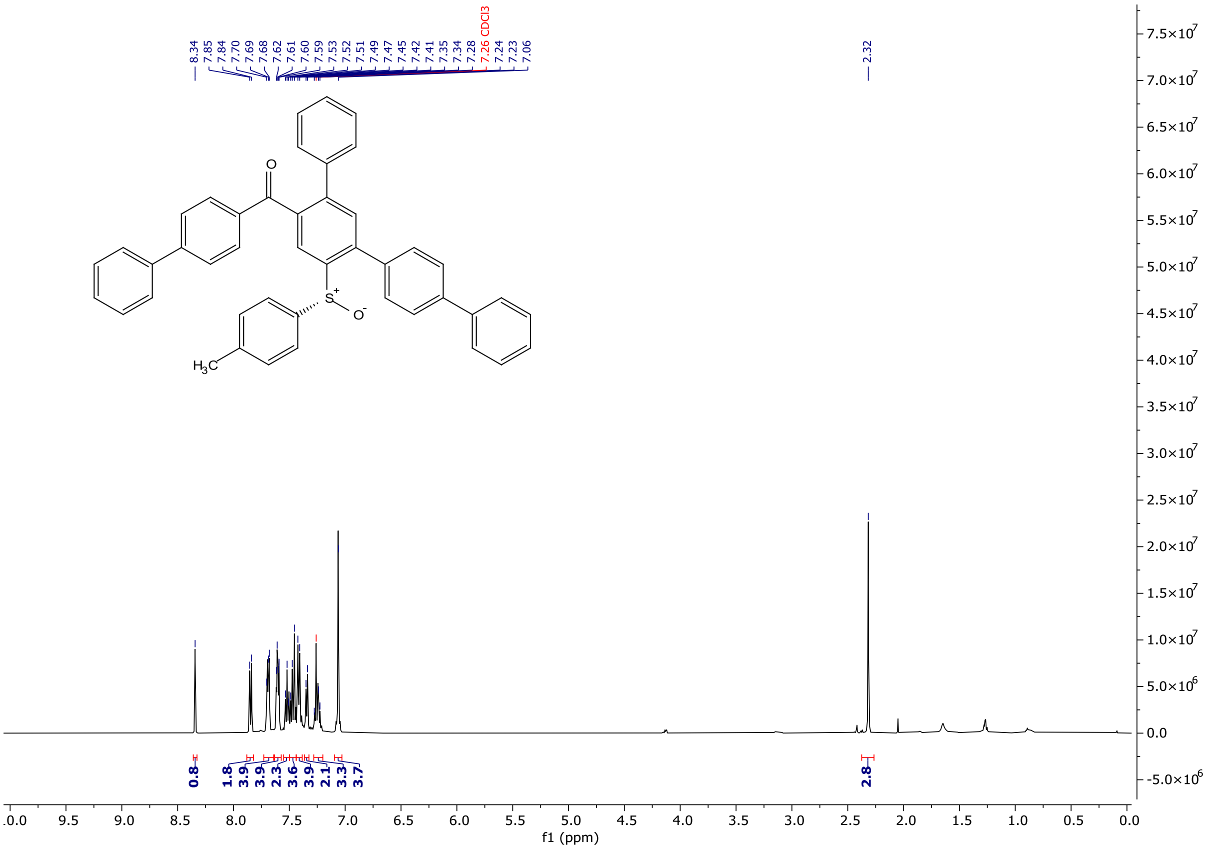

Figure S3. ^{1}H NMR (500 MHz, Chloroform-*d*) of compound **10b**.

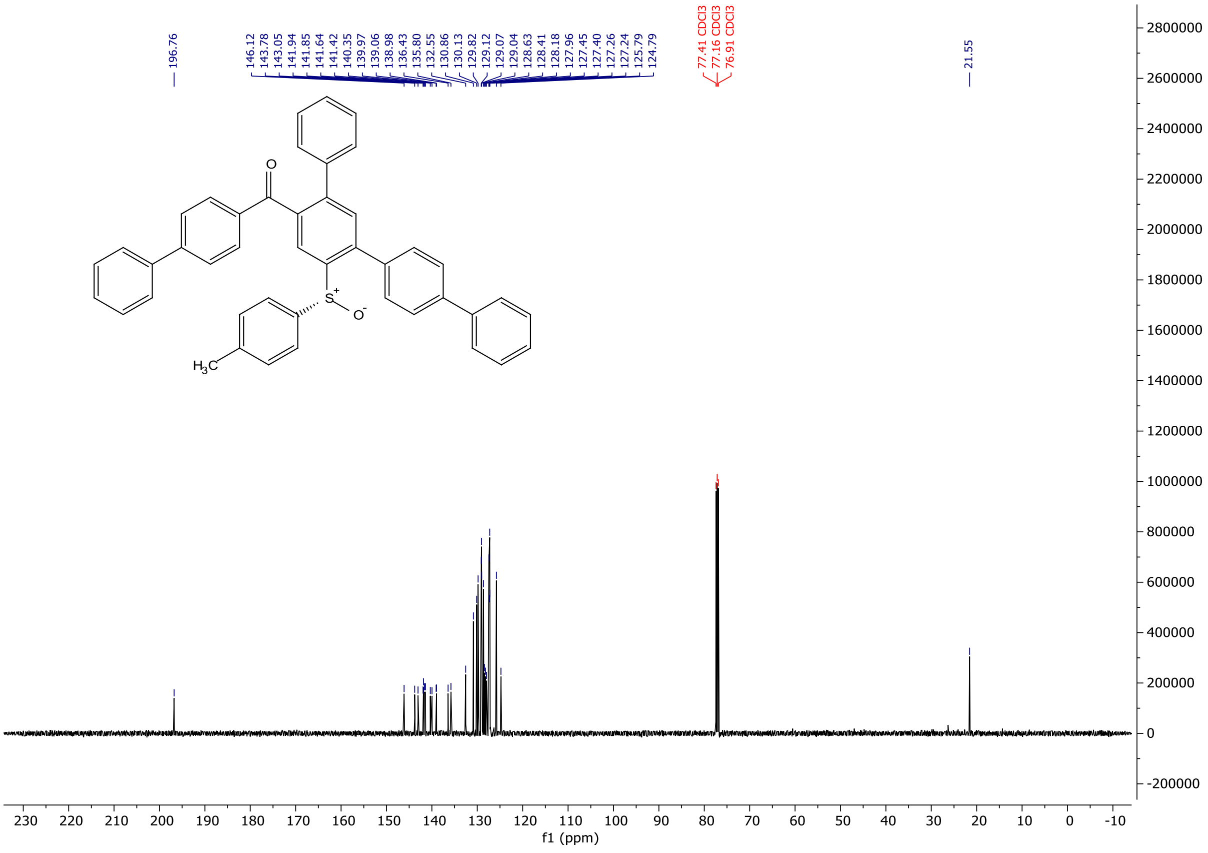

Figure S4. ^{13}C NMR (126 MHz, Chloroform-*d*) of compound **10b**.

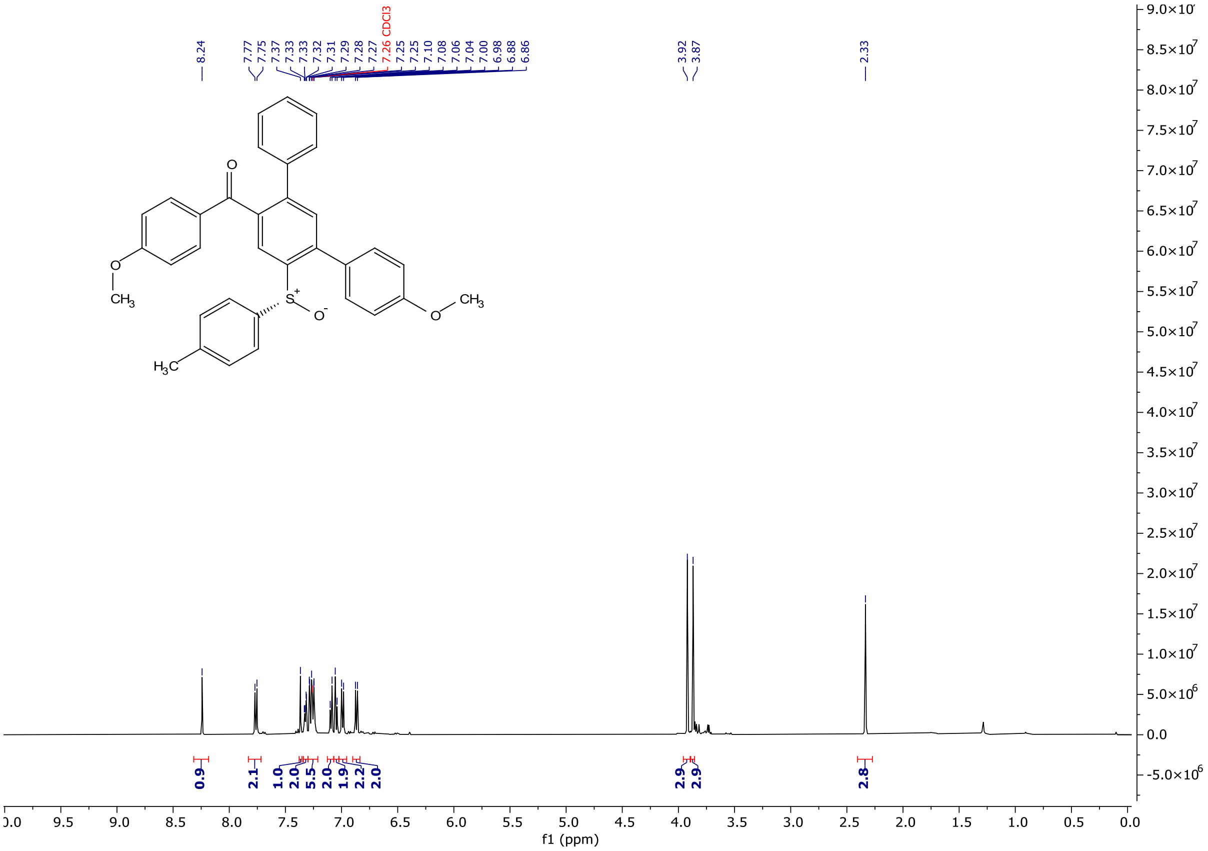

Figure S5. ^{1}H NMR (500 MHz, Chloroform-*d*) of compound **10c**.

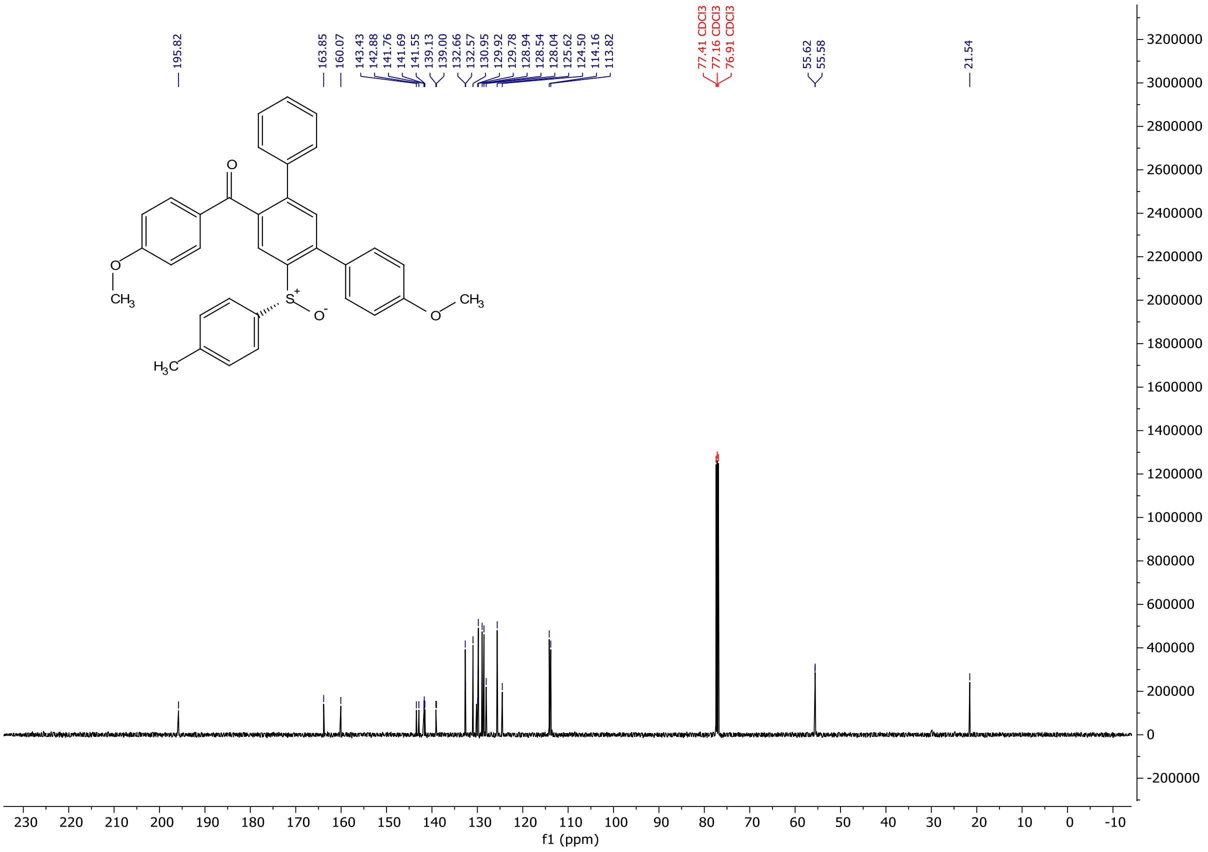

Figure S6. ^{13}C NMR (126 MHz, Chloroform-*d*) of compound **10c**.

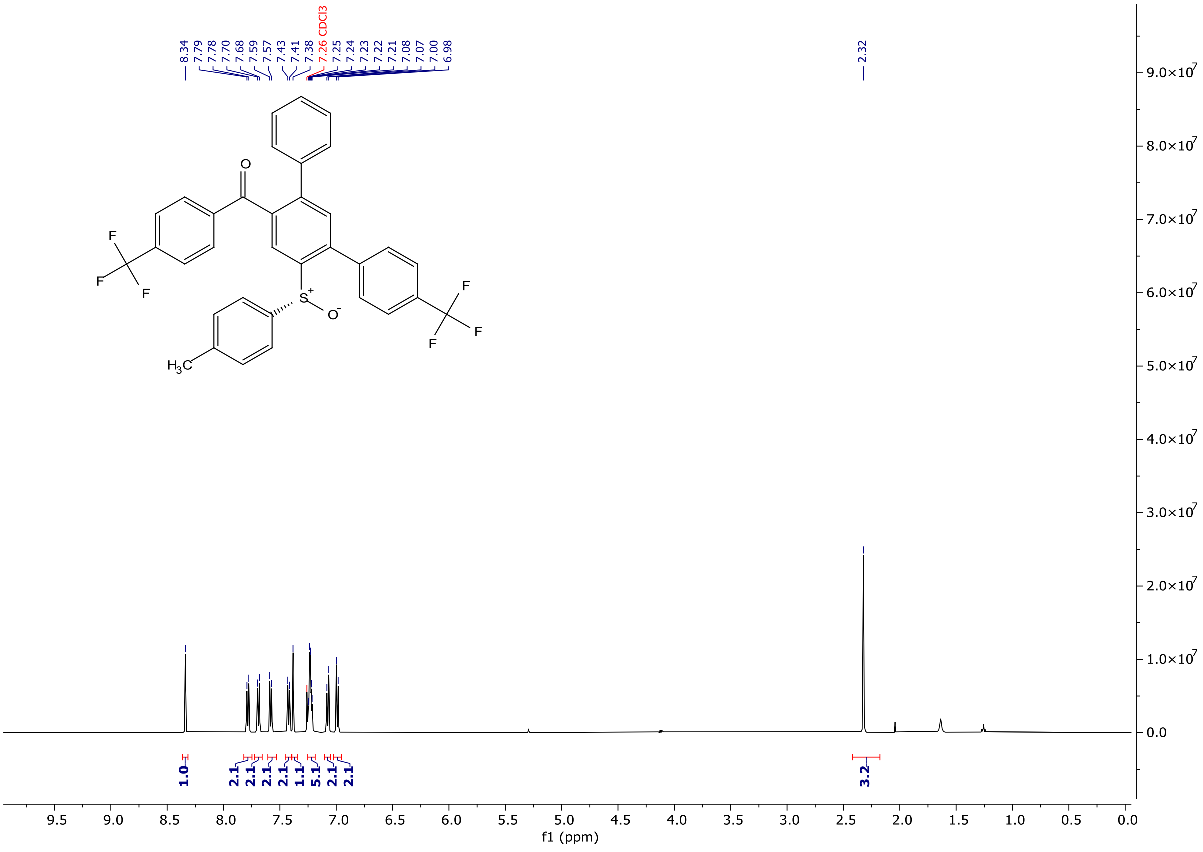

Figure S7. ^{1}H NMR (500 MHz, Chloroform-*d*) of compound **10d**.

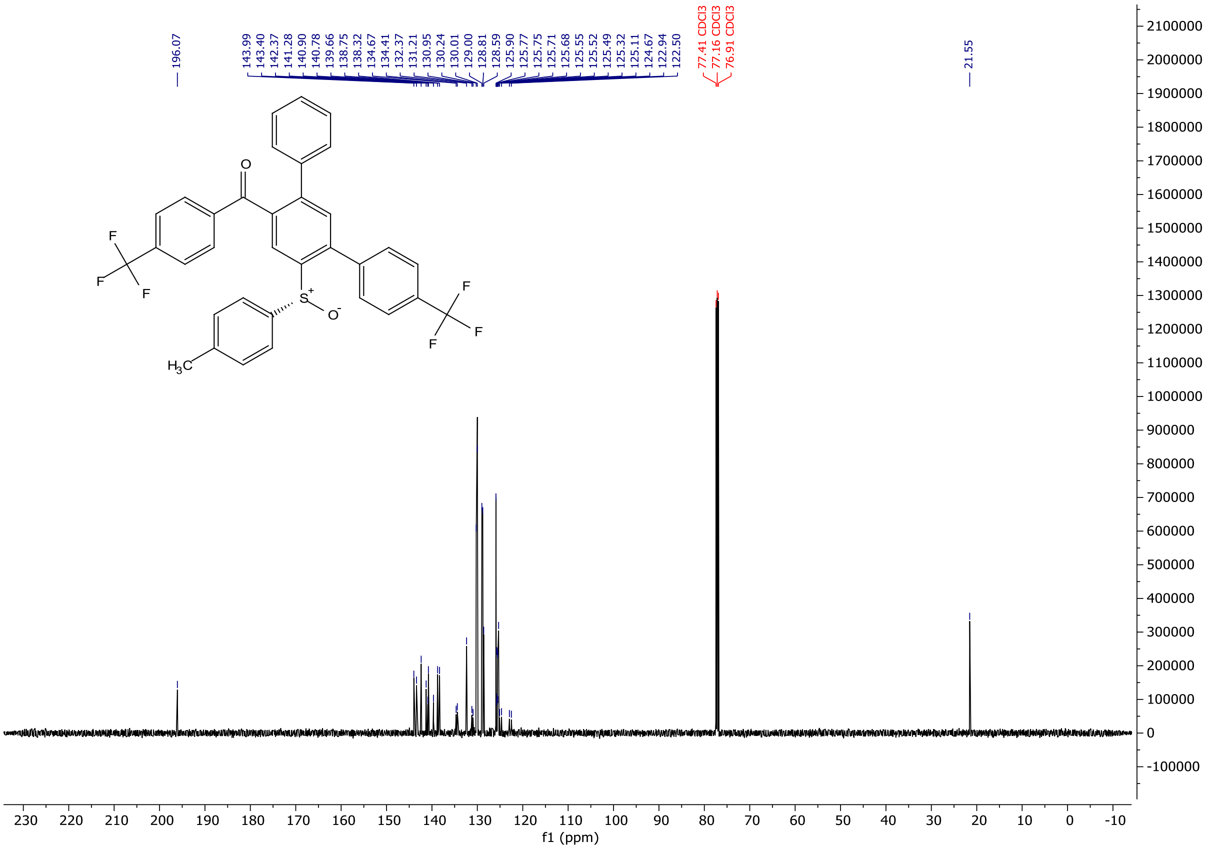

Figure S8. ^{13}C NMR (126 MHz, Chloroform-*d*) of compound **10d**.

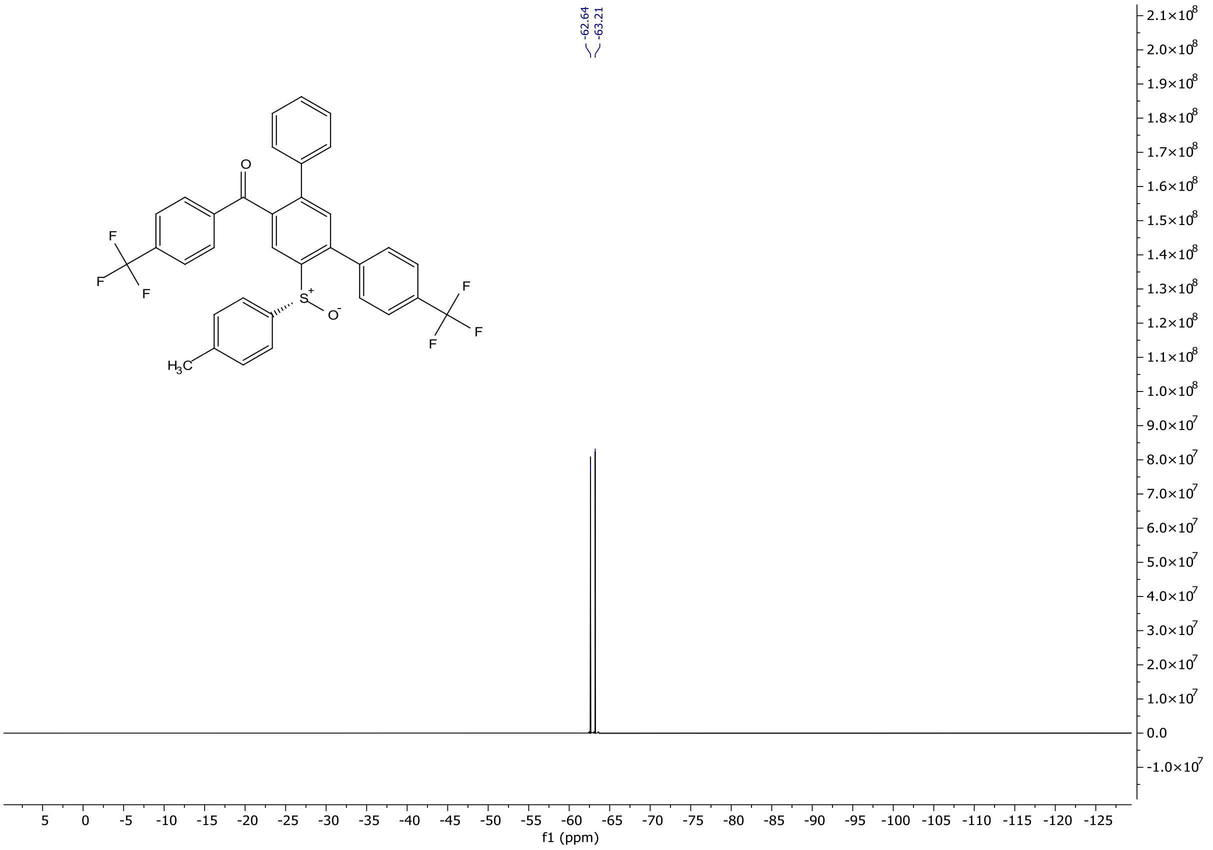

Figure S9. ^{19}F NMR (471 MHz, Chloroform-*d*) of compound **10d**.

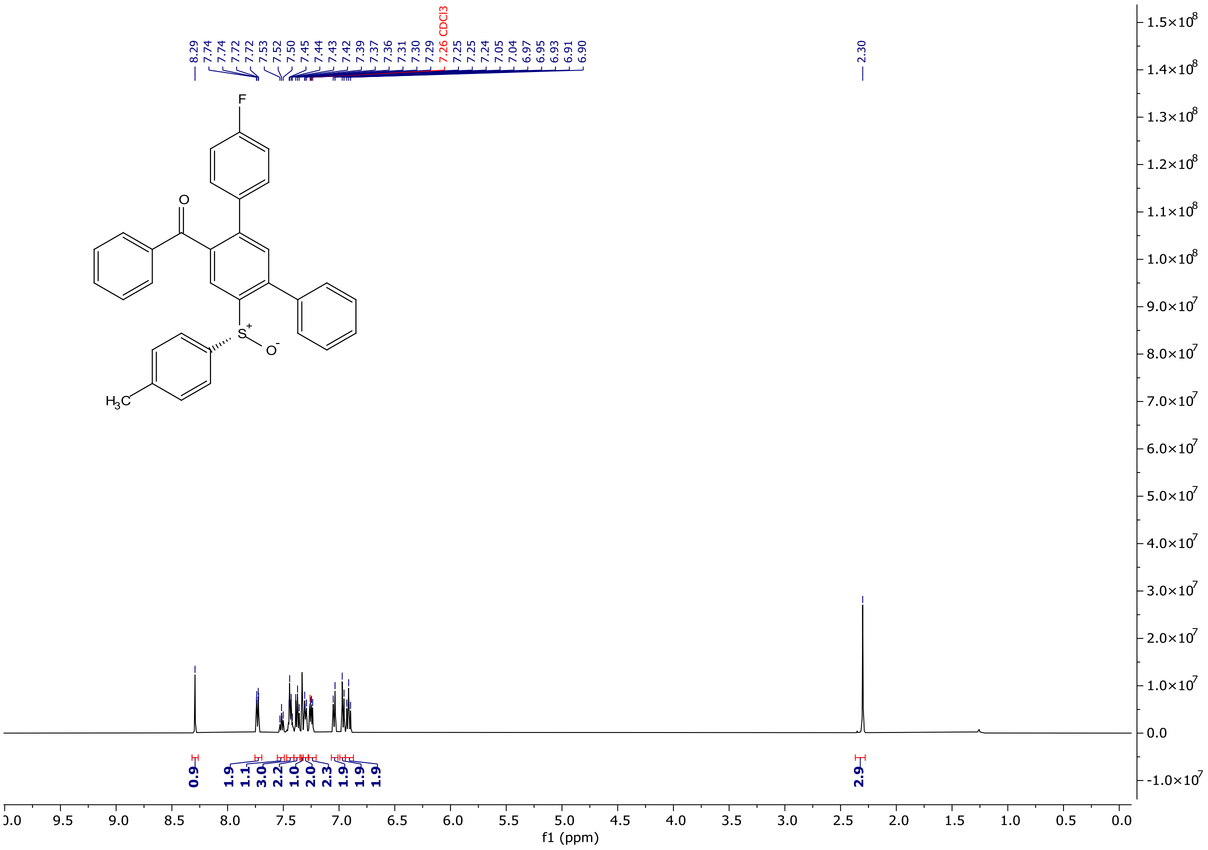

Figure S10. ^{1}H NMR (500 MHz, Chloroform-*d*) of compound **10e**.

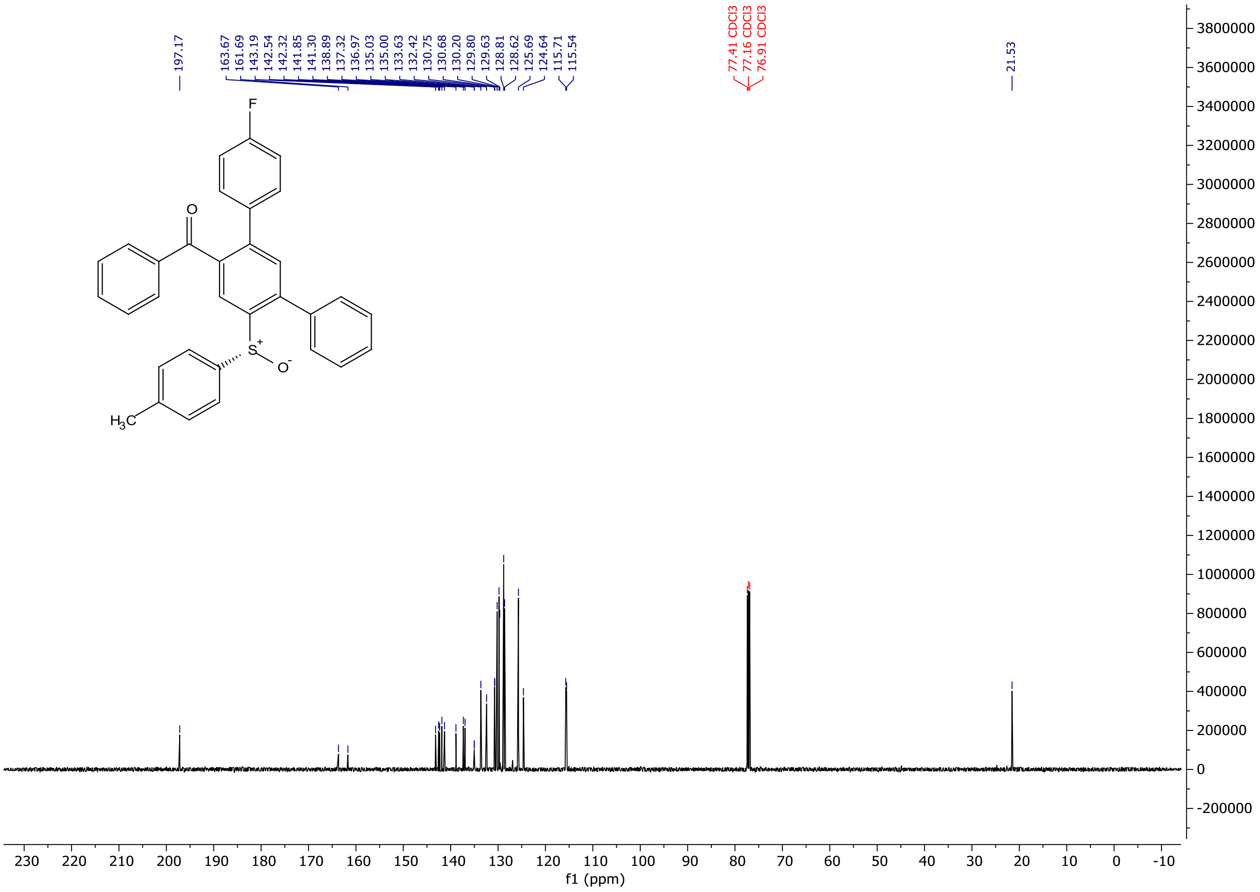

Figure S11. ^{13}C NMR (126 MHz, Chloroform-*d*) of compound **10e**.

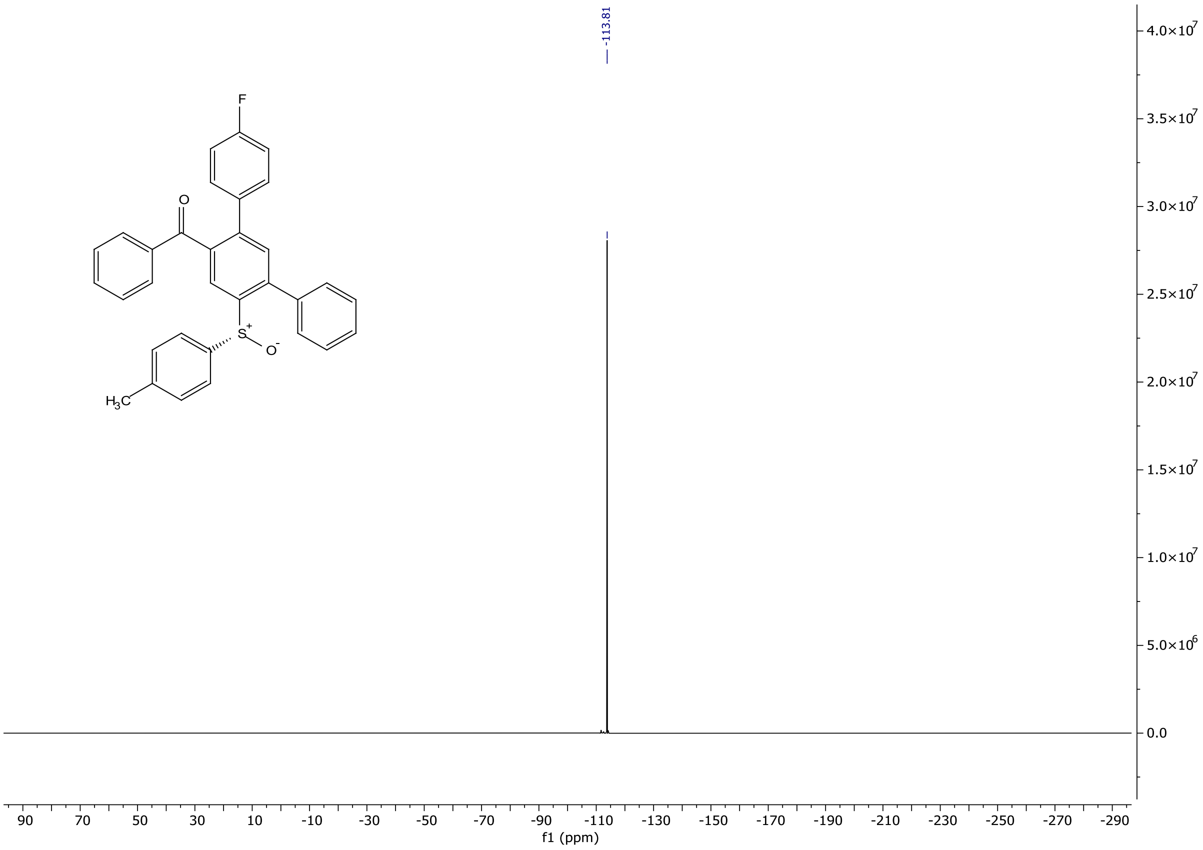

Figure S12. ^{19}F NMR (471 MHz, Chloroform-*d*) of compound **10e**.

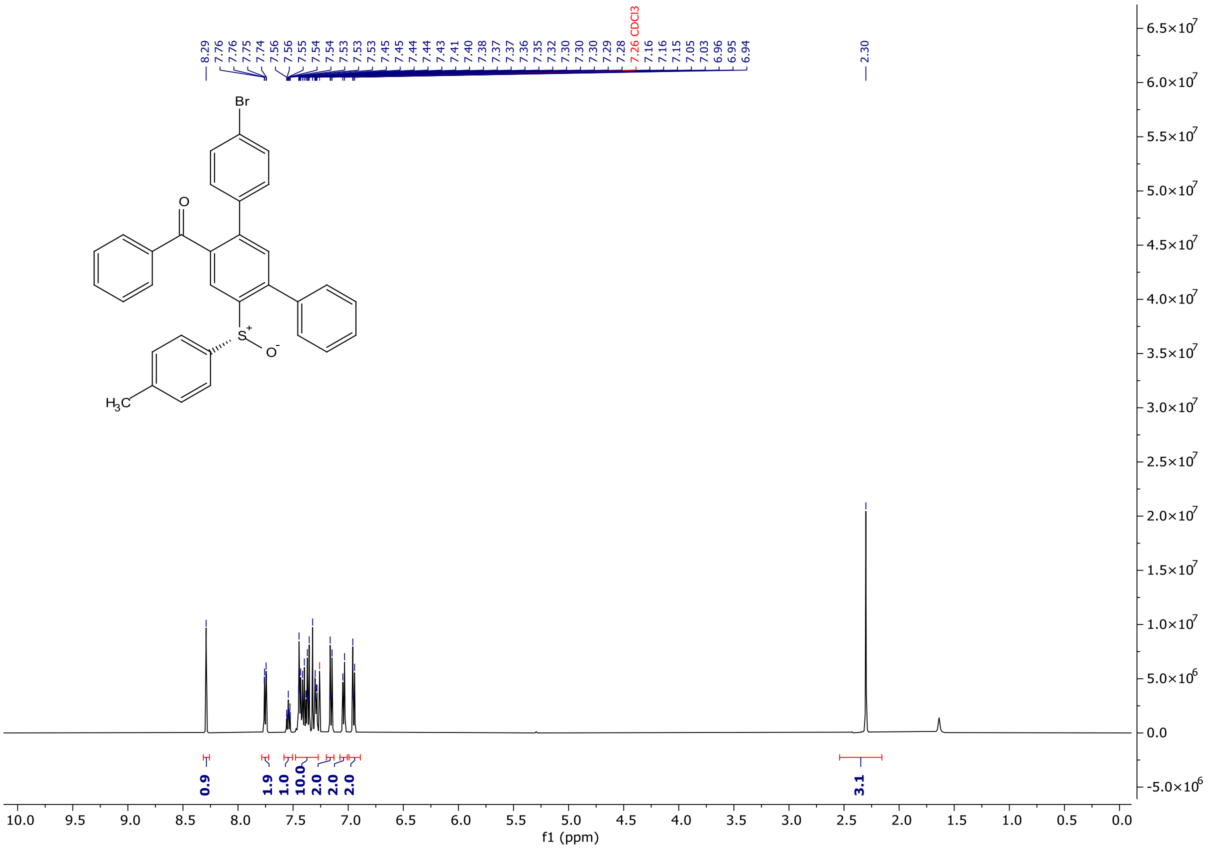

Figure S13. ^{1}H NMR (500 MHz, Chloroform-*d*) of compound **10f**.

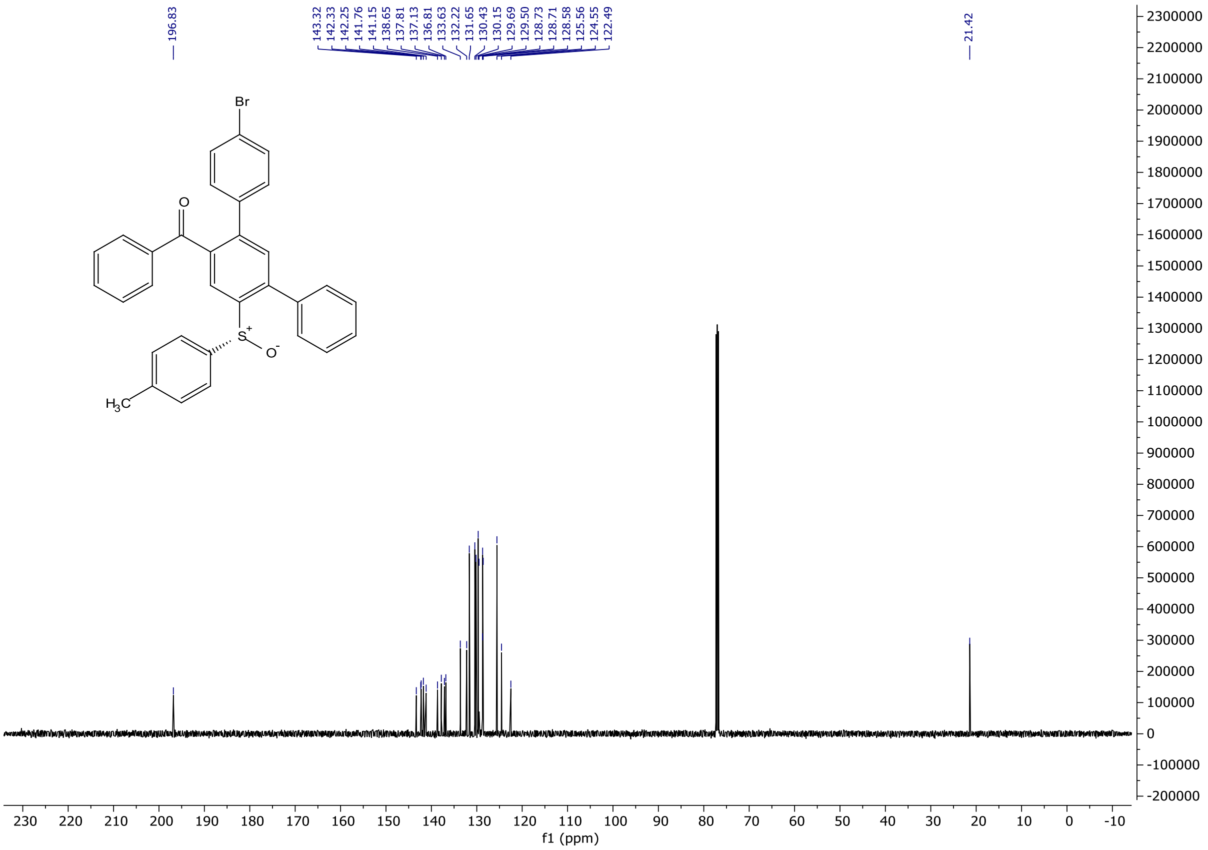

Figure S14. ^{13}C NMR (126 MHz, Chloroform-*d*) of compound **10f**.

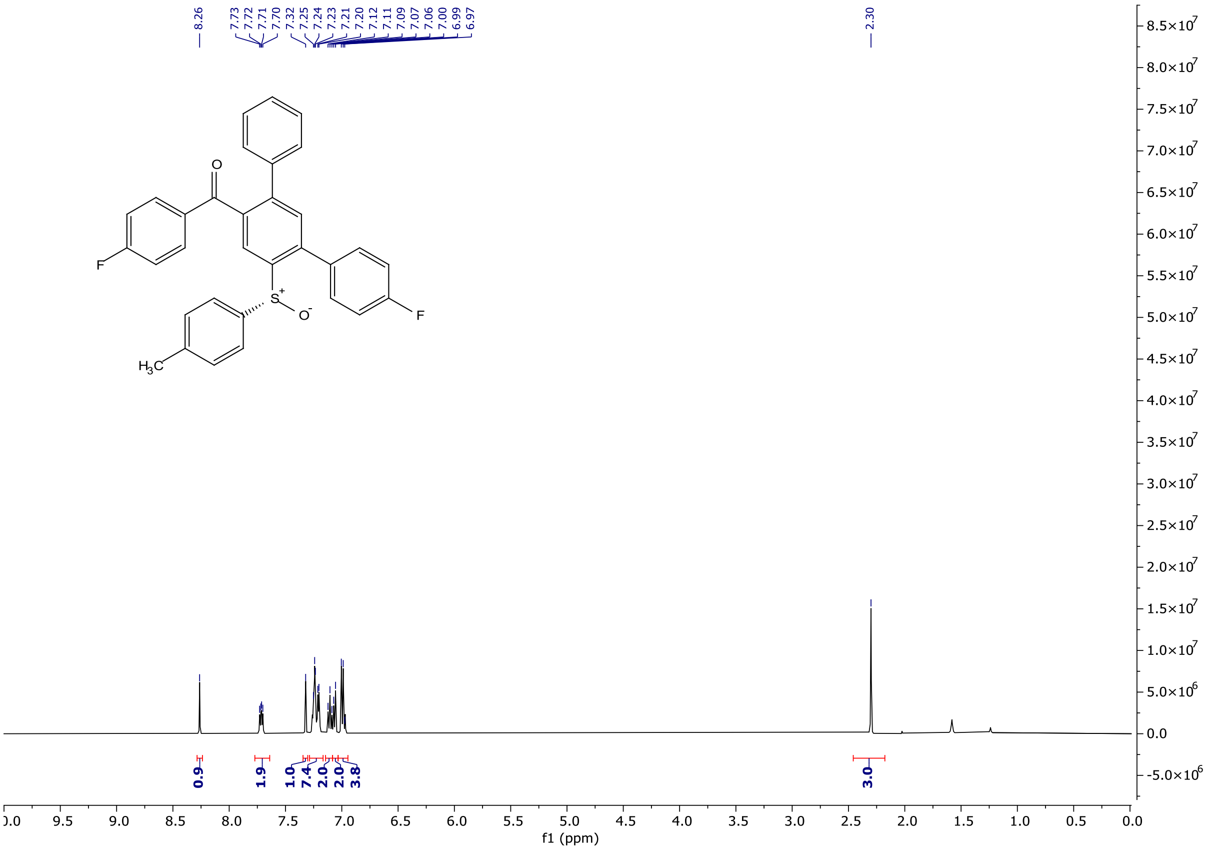

Figure S15. ^{1}H NMR (500 MHz, Chloroform-*d*) of compound **10g**.

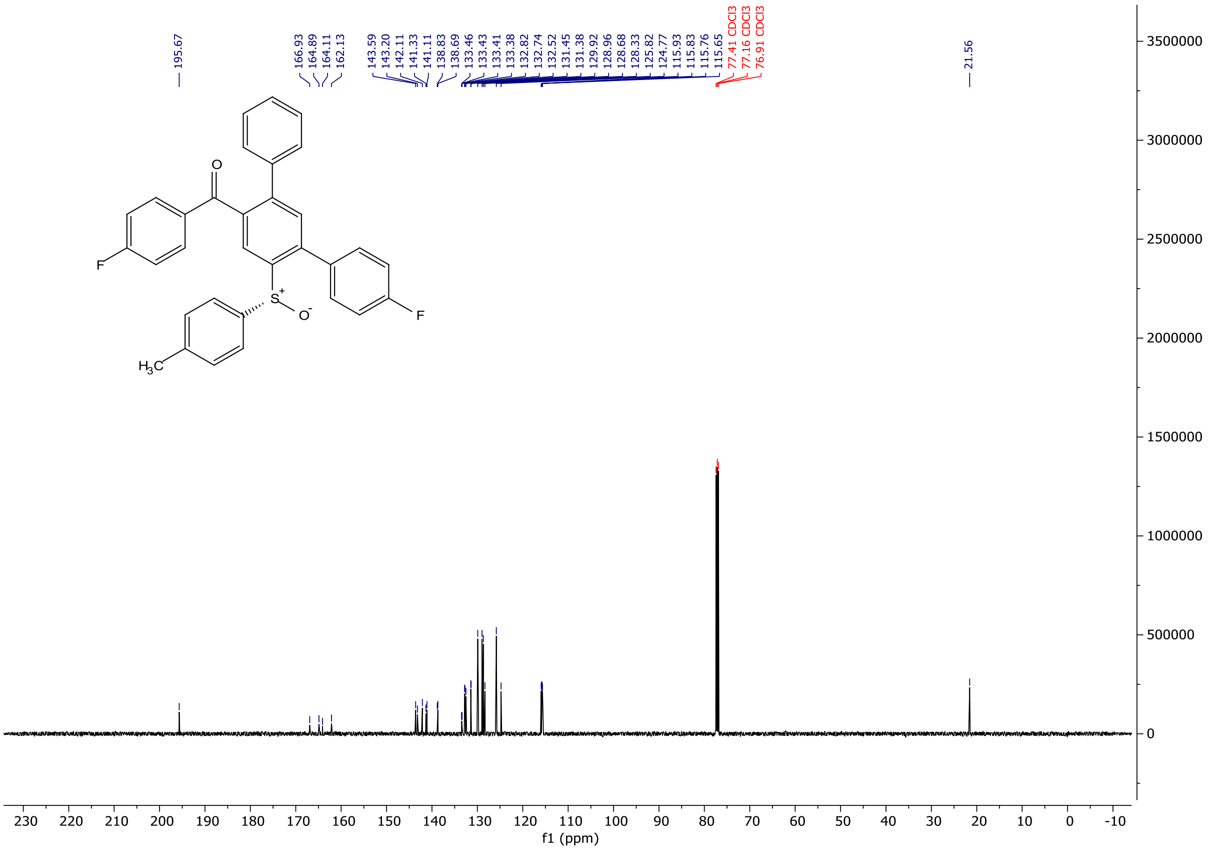

Figure S16. ^{13}C NMR (126 MHz, Chloroform-*d*) of compound **10g**.

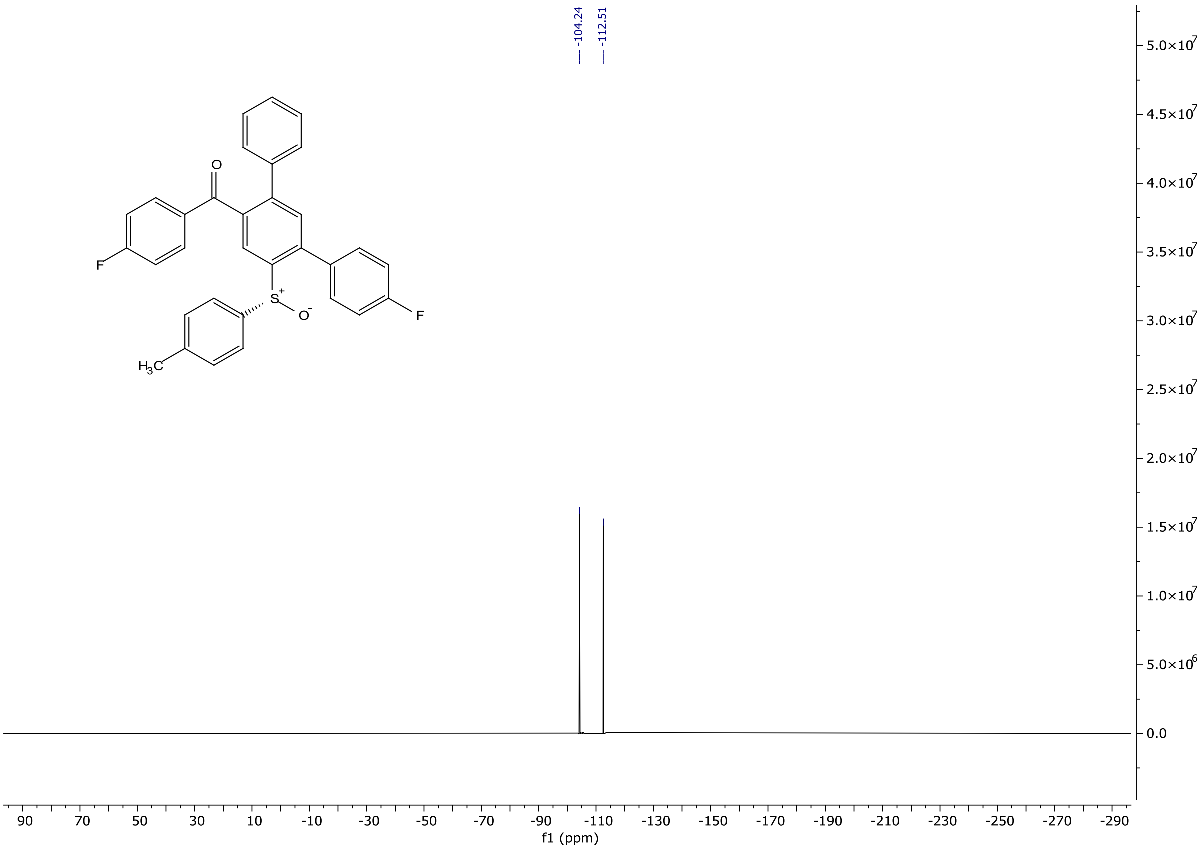

Figure S17. ^{19}F NMR (471 MHz, Chloroform-*d*) of compound **10g**.

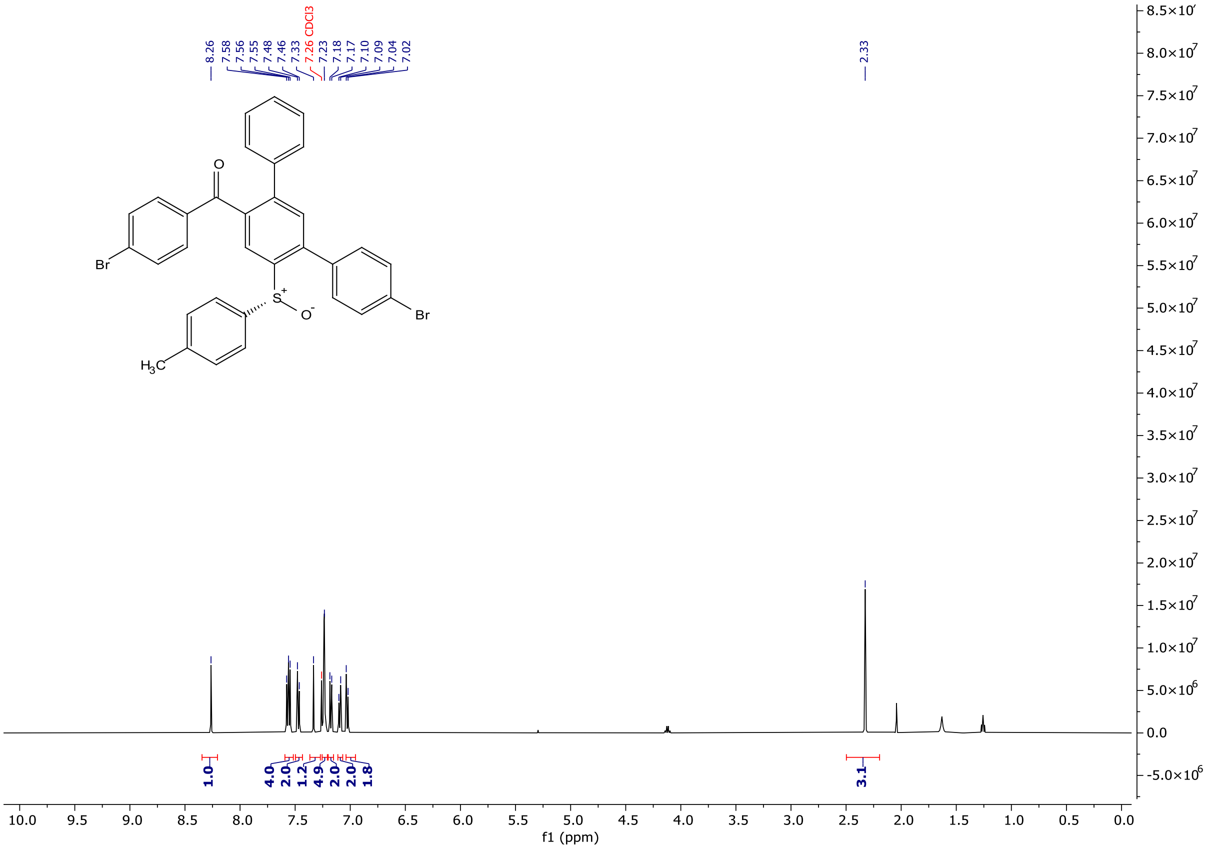

Figure S18. ^{1}H NMR (500 MHz, Chloroform-*d*) of compound **10h**.

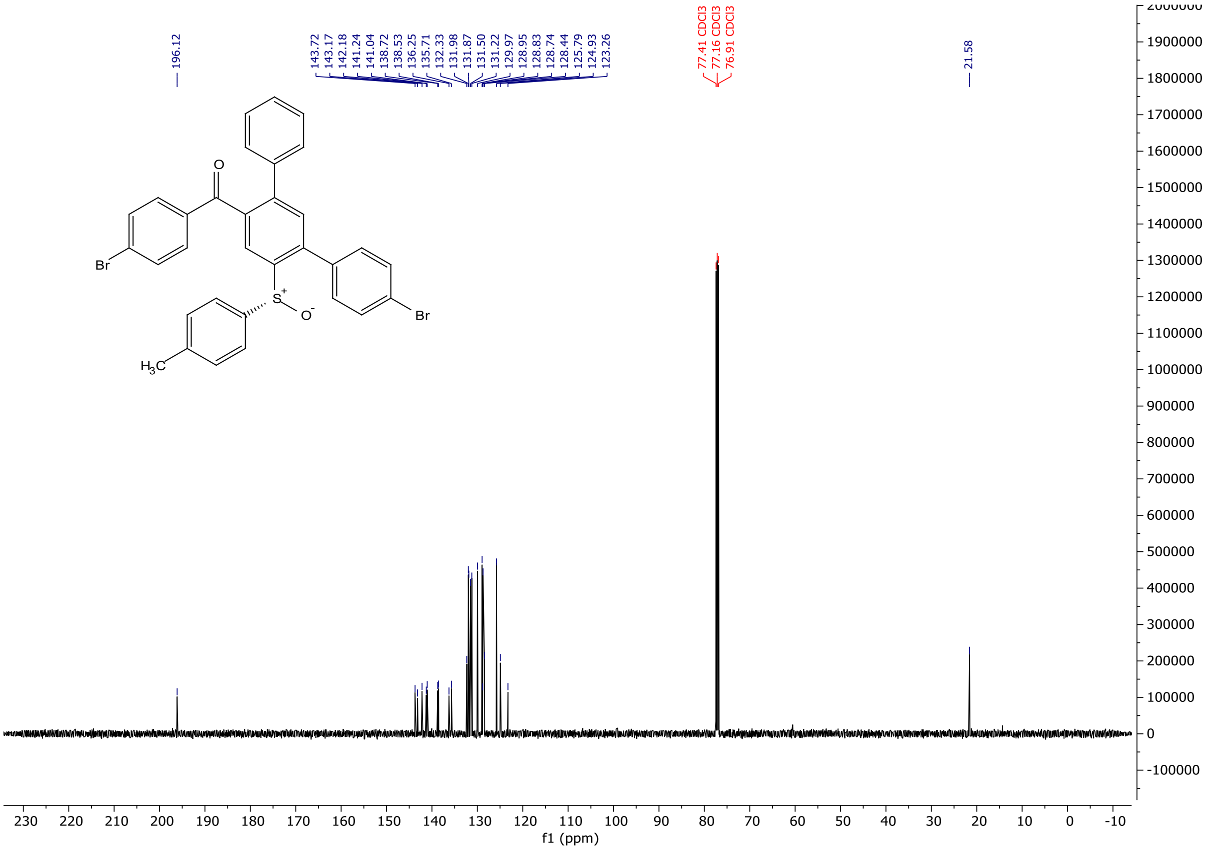

Figure S19. ^{13}C NMR (126 MHz, Chloroform-*d*) of compound **10h**.

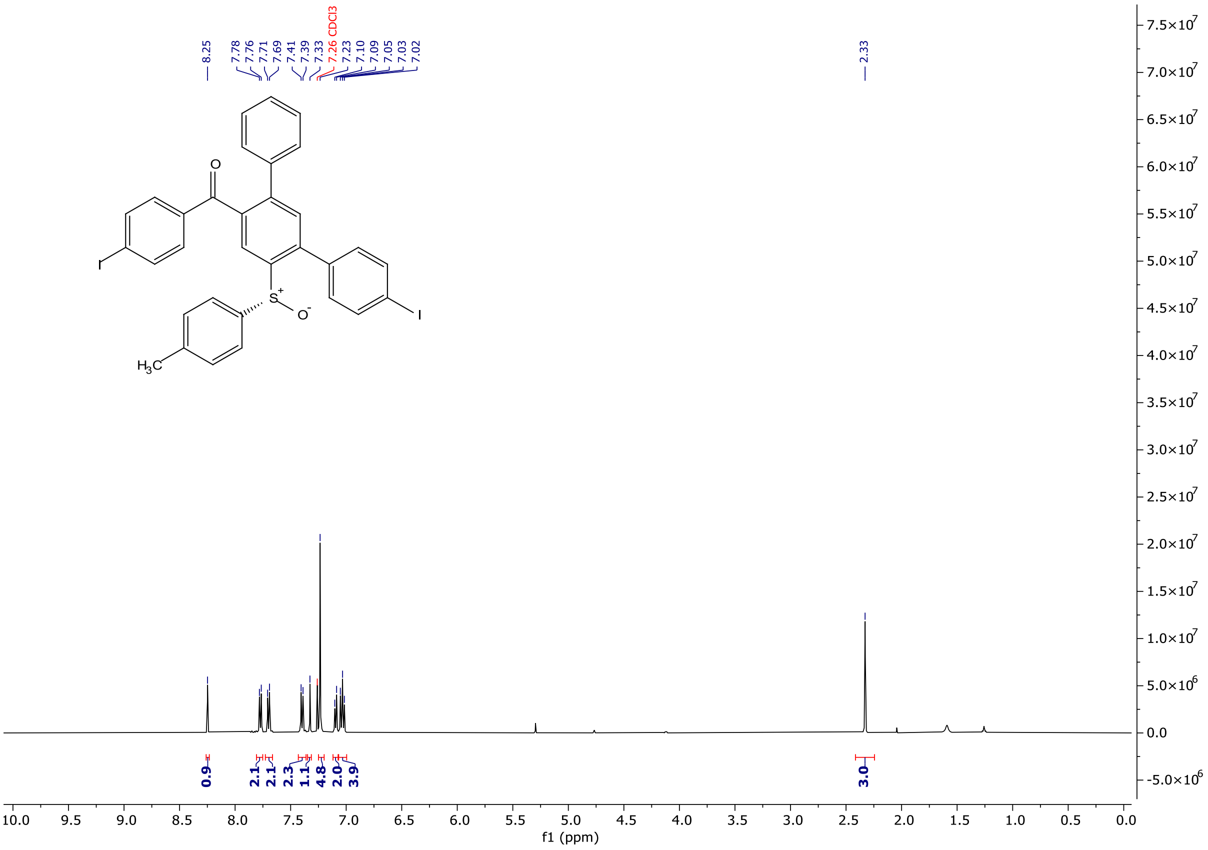

Figure S20. ^{1}H NMR (500 MHz, Chloroform-*d*) of compound **10i**.

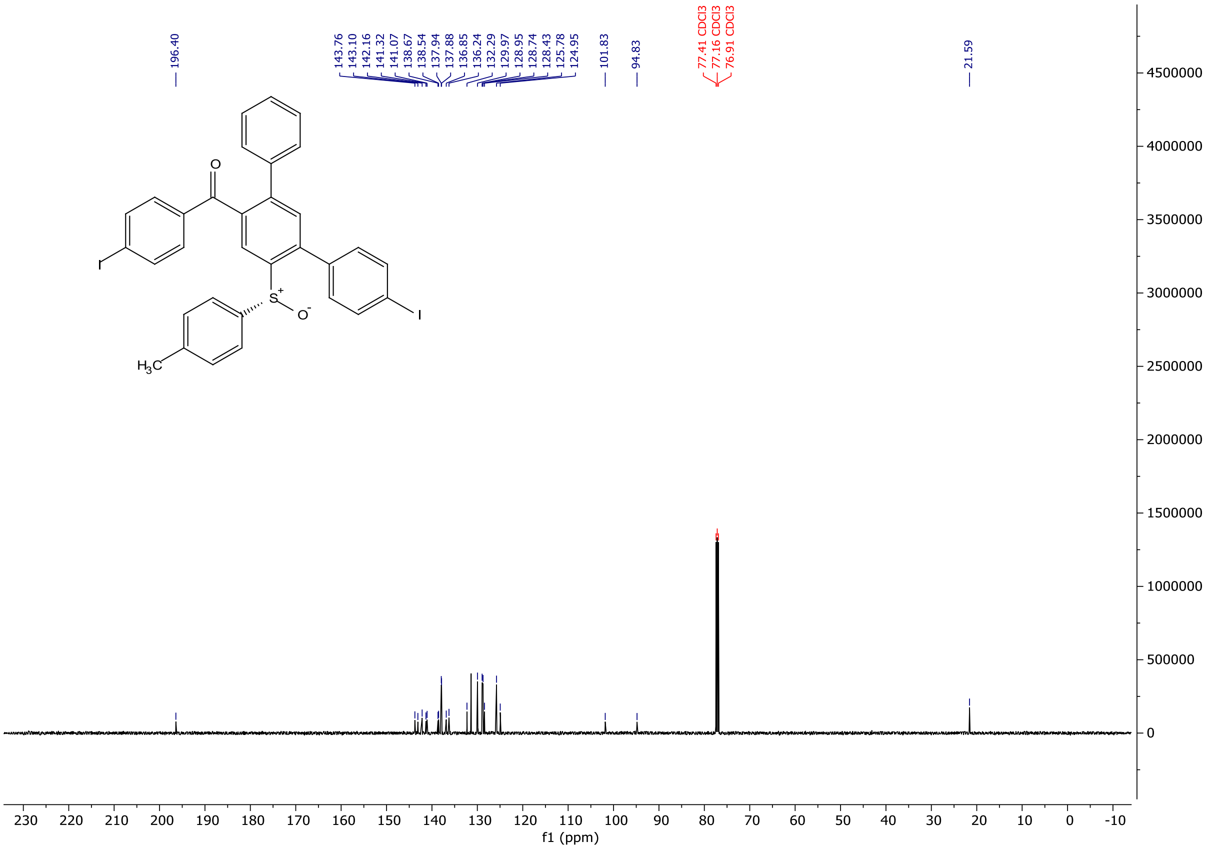

Figure S21. ^{13}C NMR (126 MHz, Chloroform-*d*) of compound **10i**.

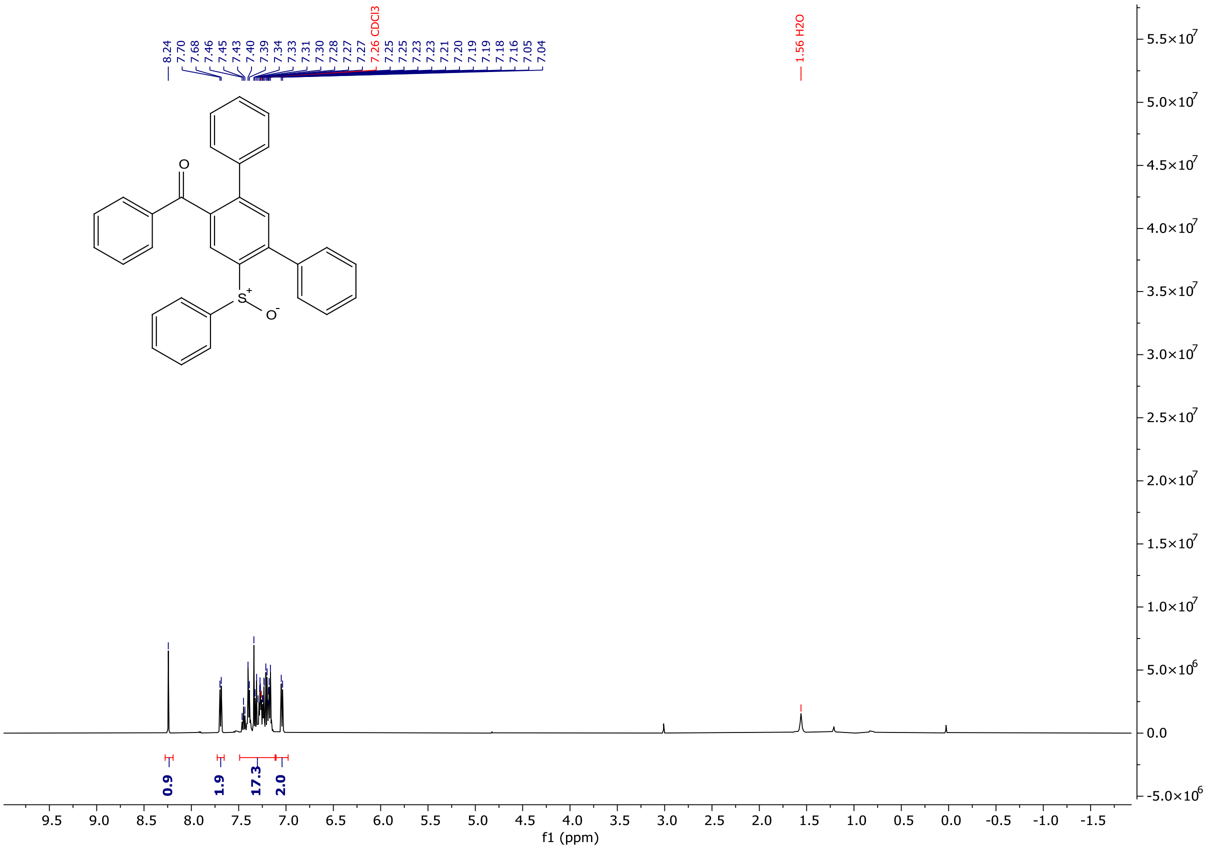

Figure S22. ^{1}H NMR (500 MHz, Chloroform-*d*) of compound **10j**.

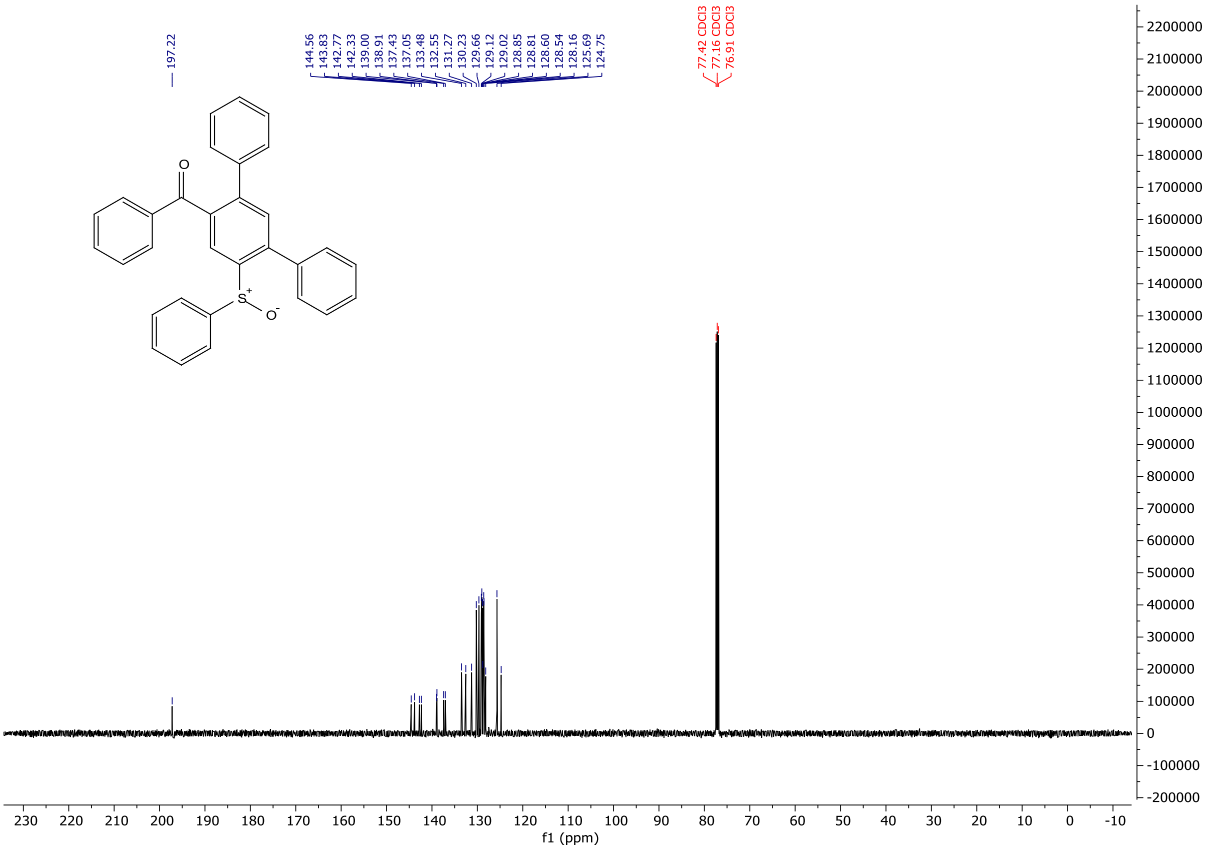

Figure S23. ^{13}C NMR (126 MHz, Chloroform-*d*) of compound **10j**.

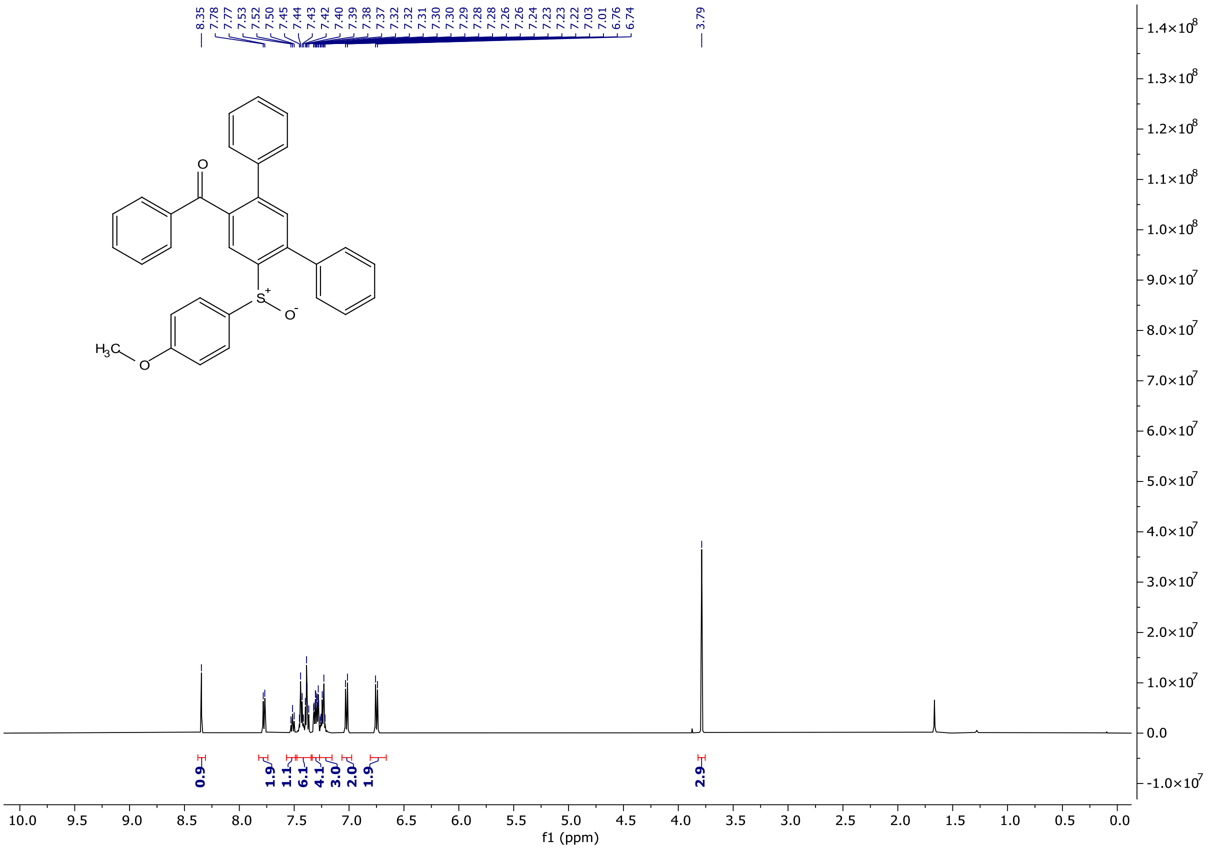

Figure S24. ^{1}H NMR (500 MHz, Chloroform-*d*) of compound **10k**.

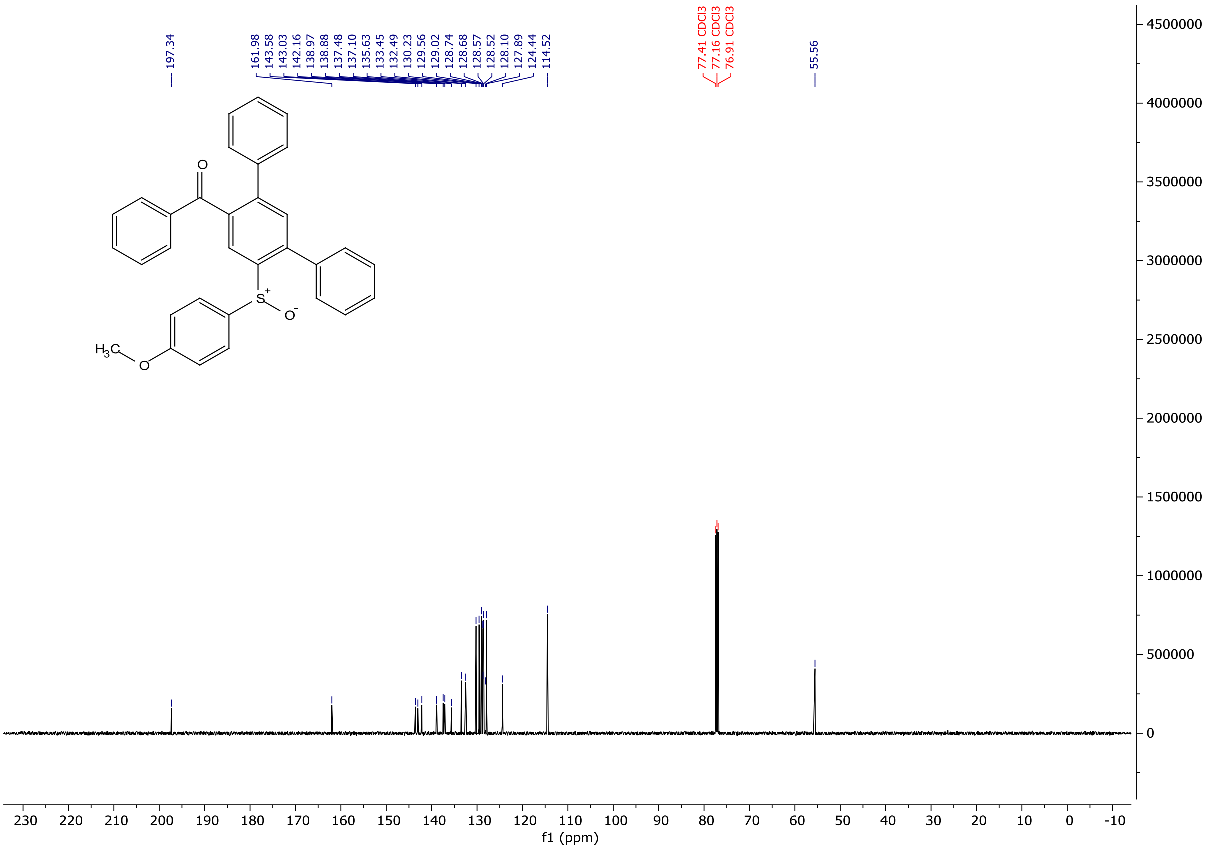

Figure S25. ^{13}C NMR (126 MHz, Chloroform-*d*) of compound **10k**.

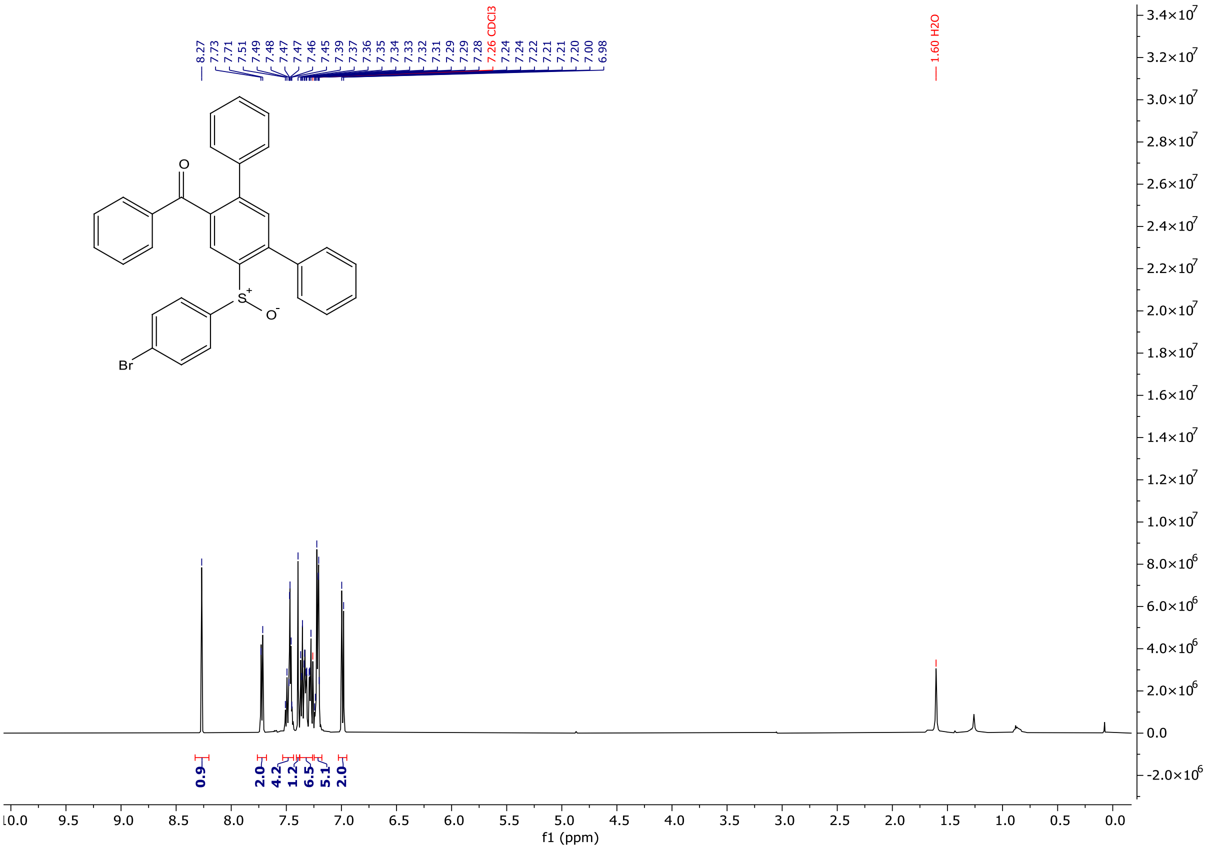

Figure S26. ^{1}H NMR (500 MHz, Chloroform-*d*) of compound **10l**.

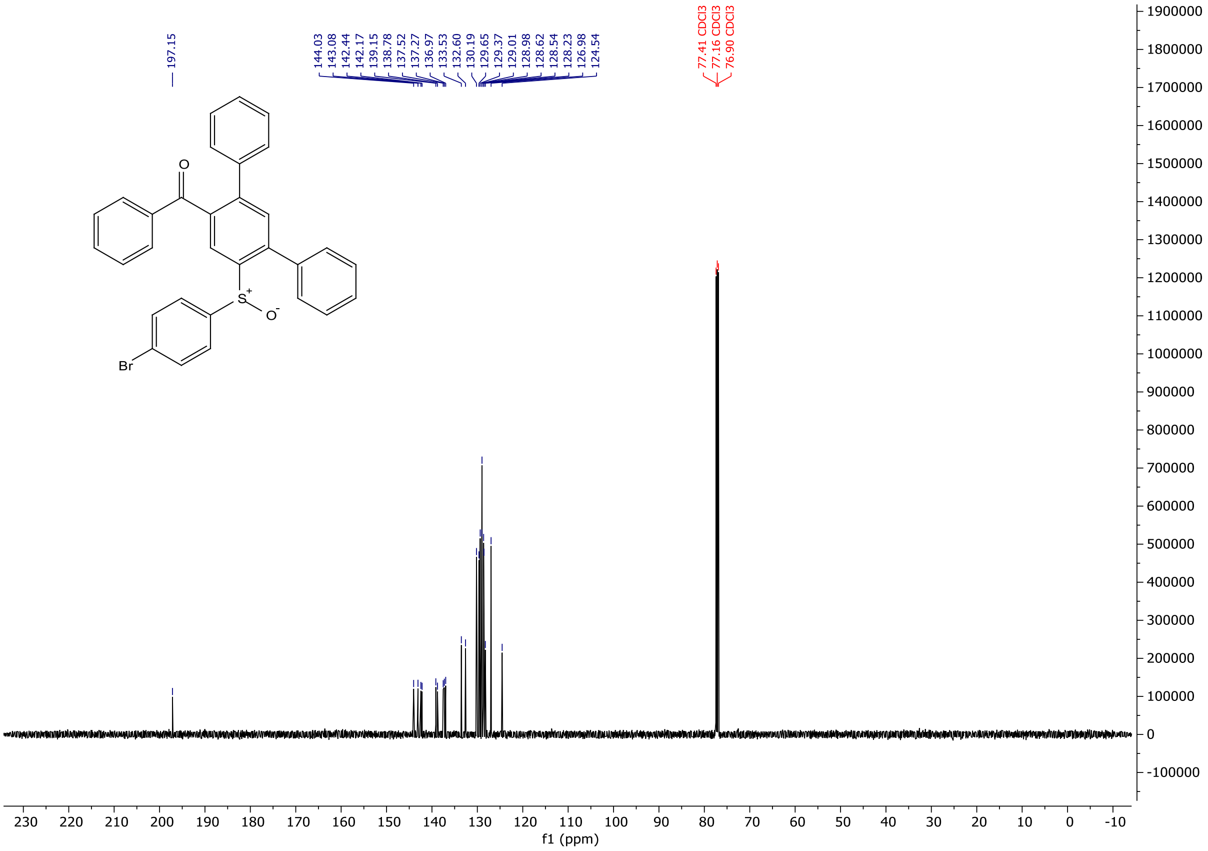

Figure S27. ^{13}C NMR (126 MHz, Chloroform-*d*) of compound **10l**.

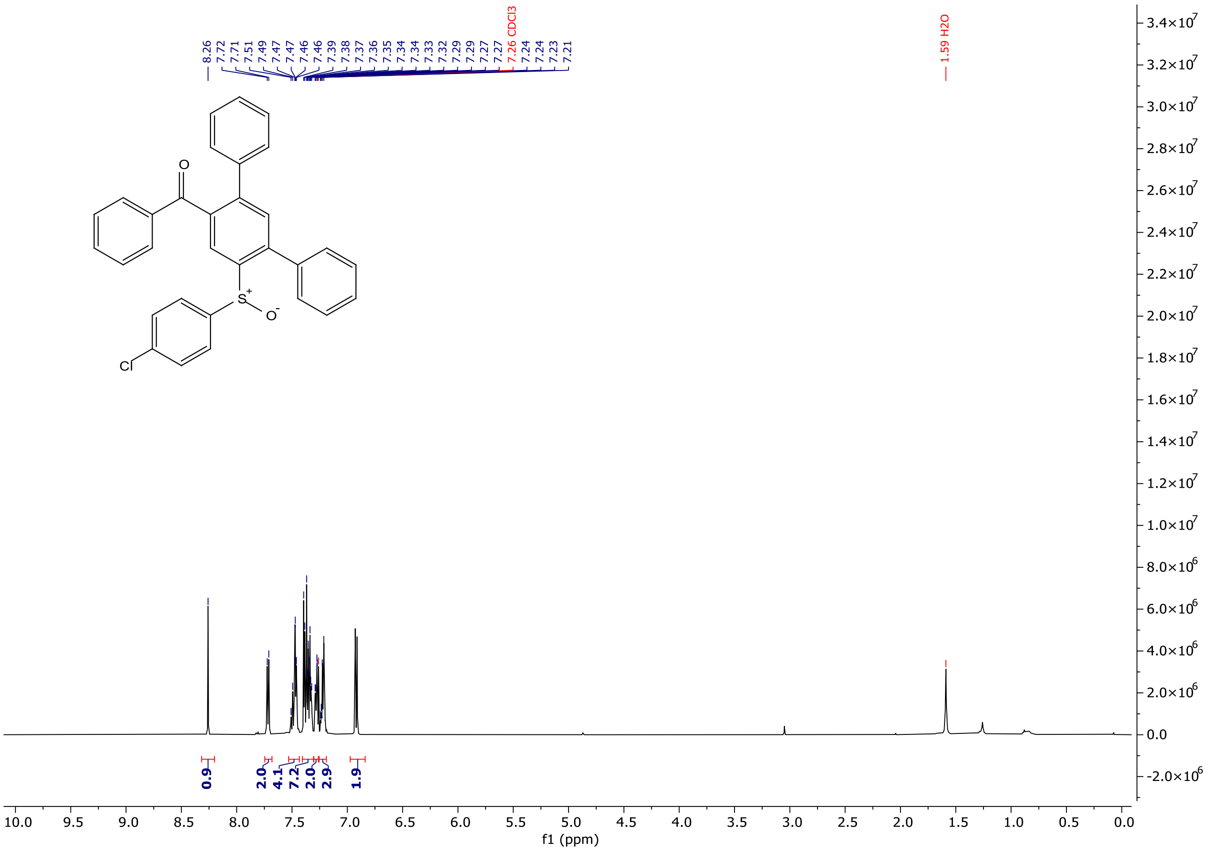

Figure S28. ^{1}H NMR (500 MHz, Chloroform-*d*) of compound **10m**.

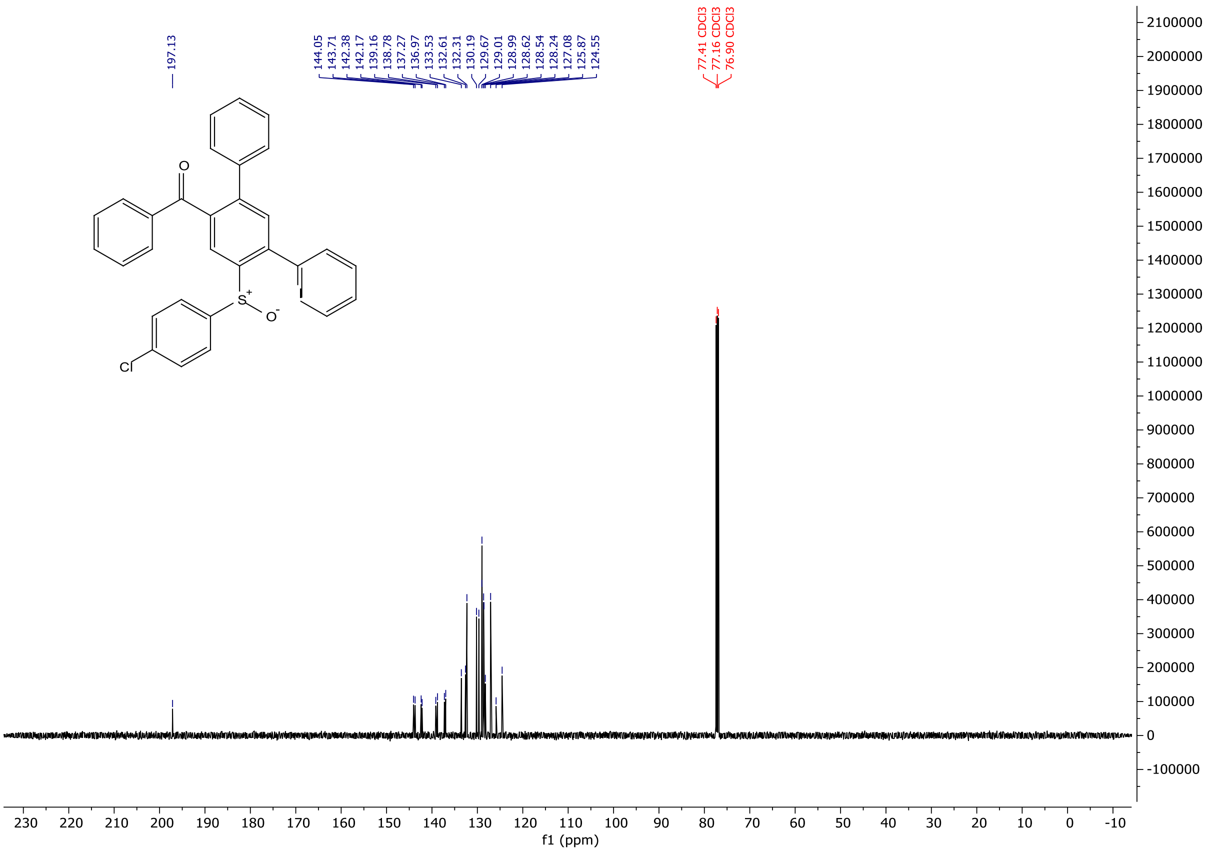

Figure S29. ^{13}C NMR (126 MHz, Chloroform-*d*) of compound **10m**.

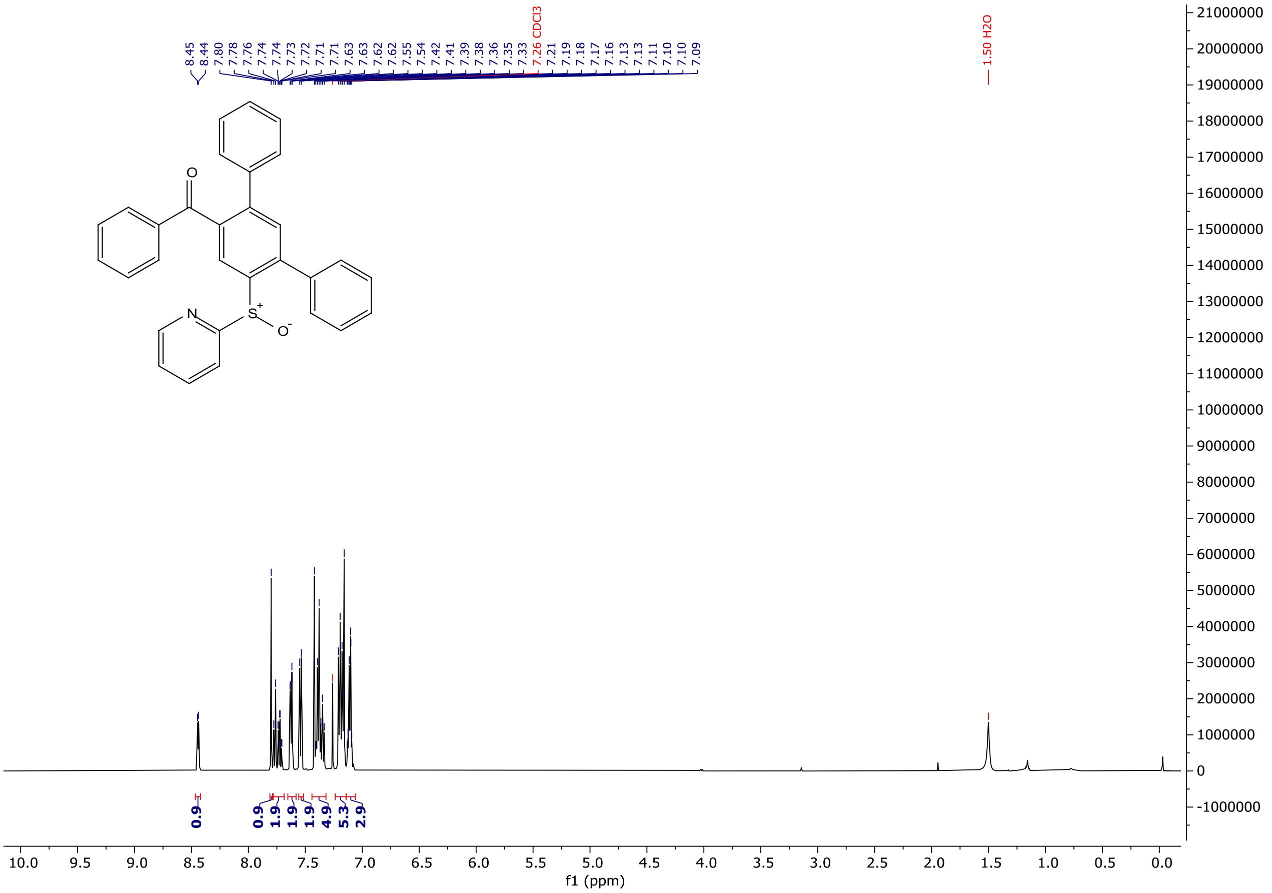

Figure S30. ^{1}H NMR (500 MHz, Chloroform-*d*) of compound **10n**.

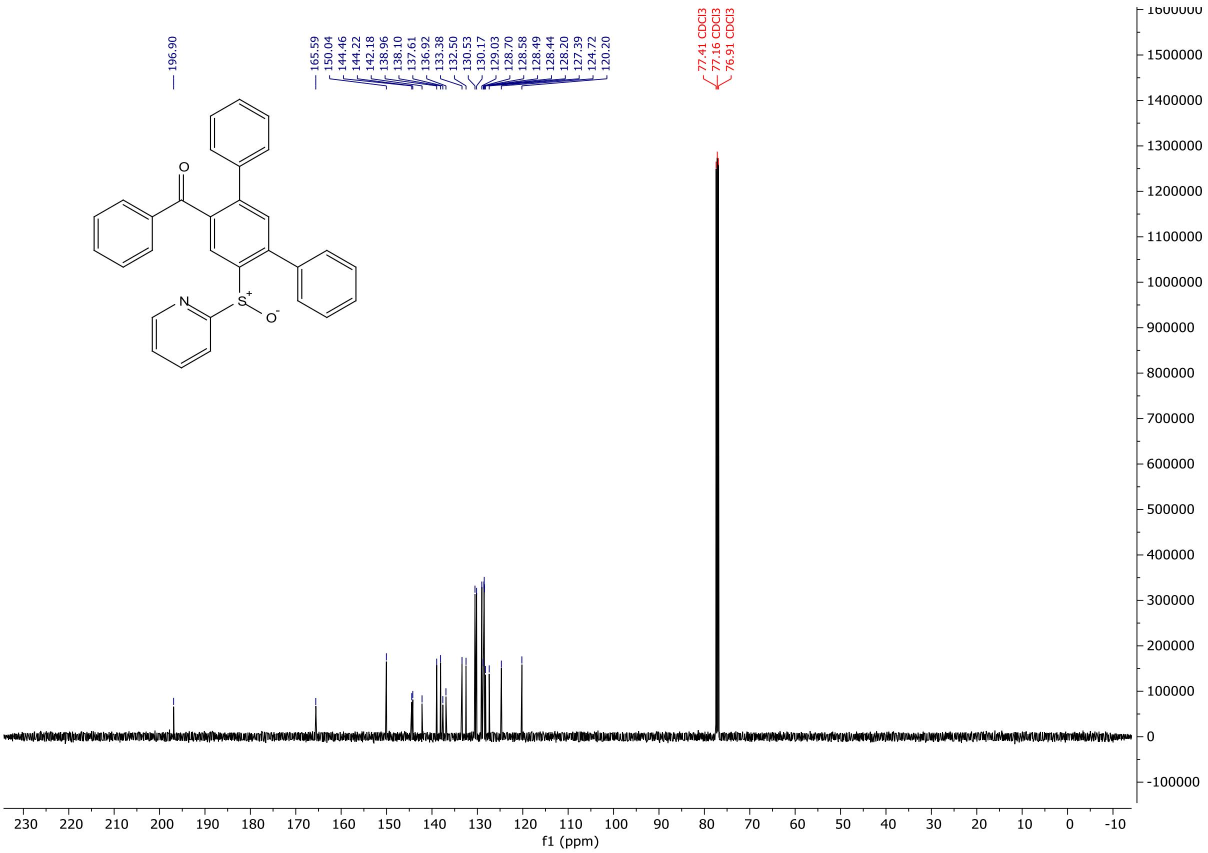

Figure S31. ^{13}C NMR (126 MHz, Chloroform-*d*) of compound **10n**.

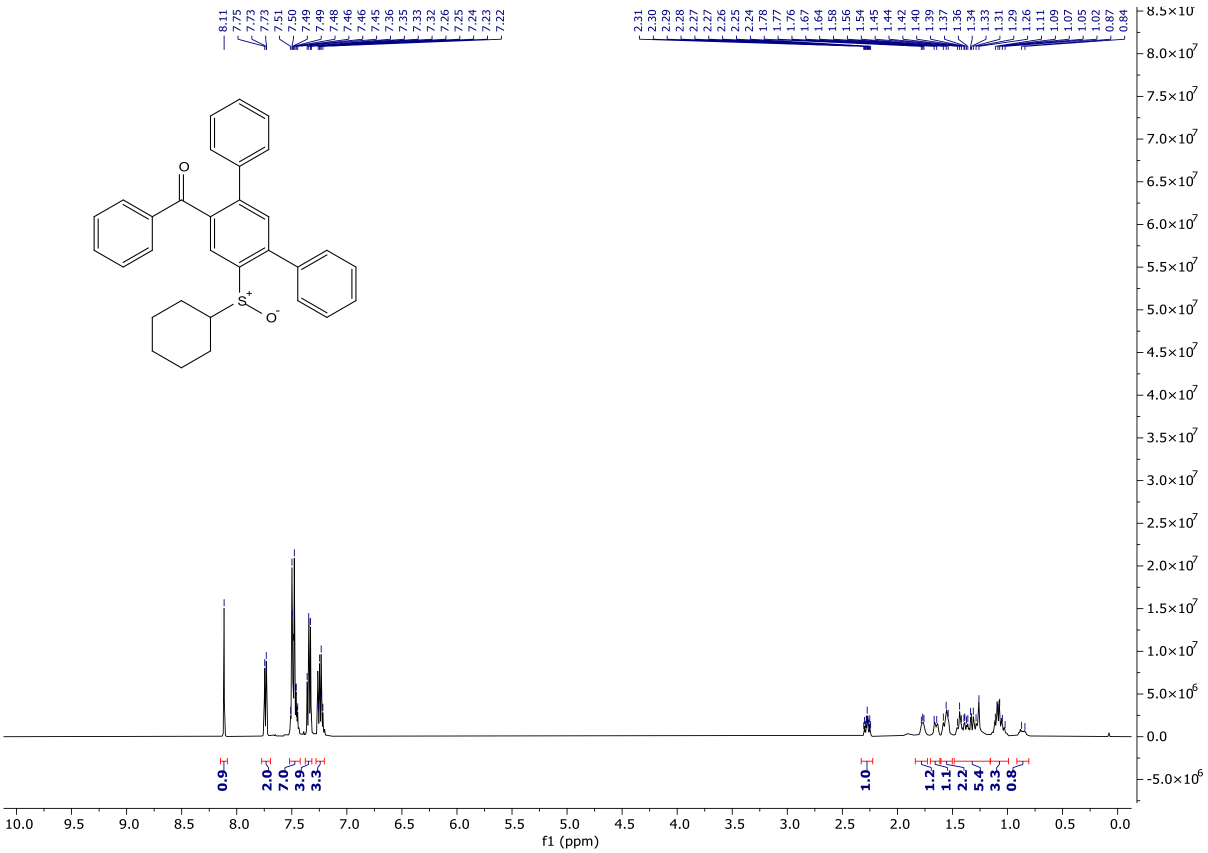

Figure S32. ^{1}H NMR (500 MHz, Chloroform-*d*) of compound **10o**.

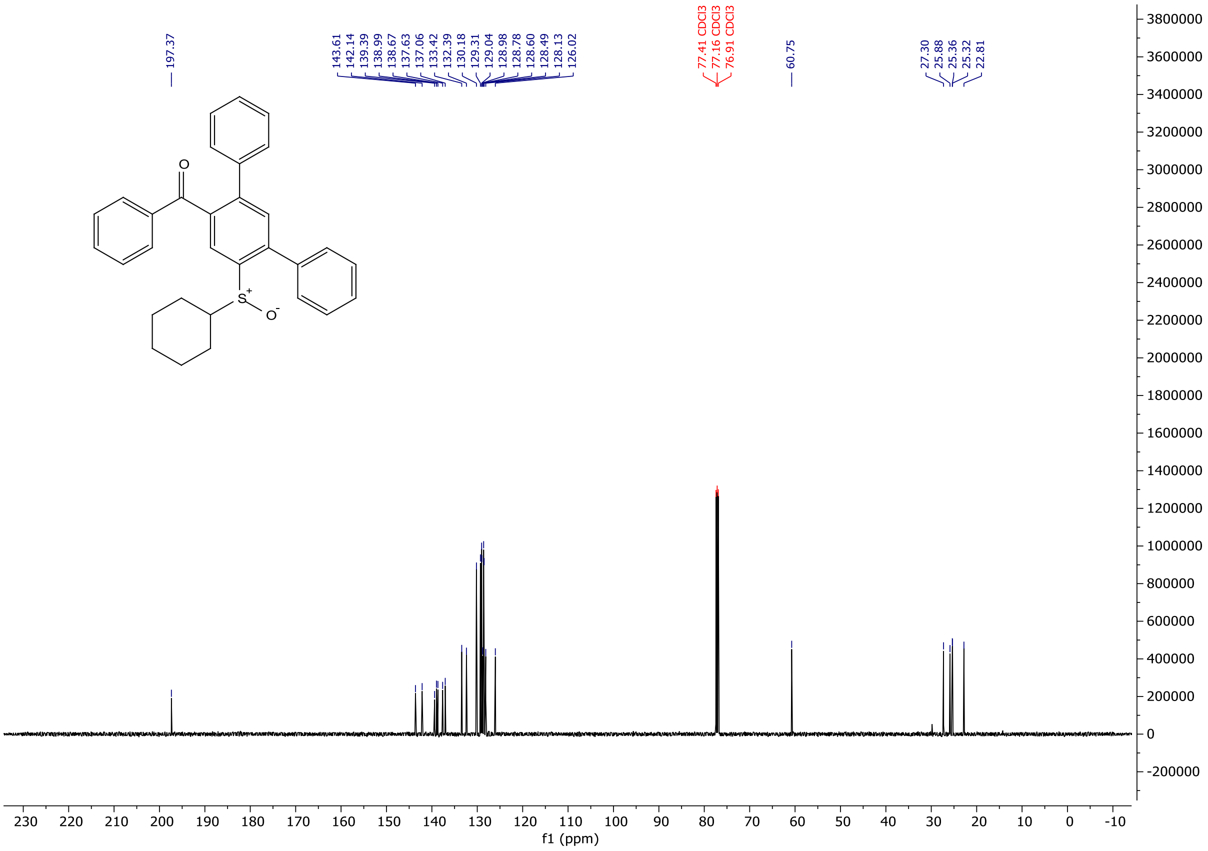

Figure S33. ^{13}C NMR (126 MHz, Chloroform-*d*) of compound **10o**.

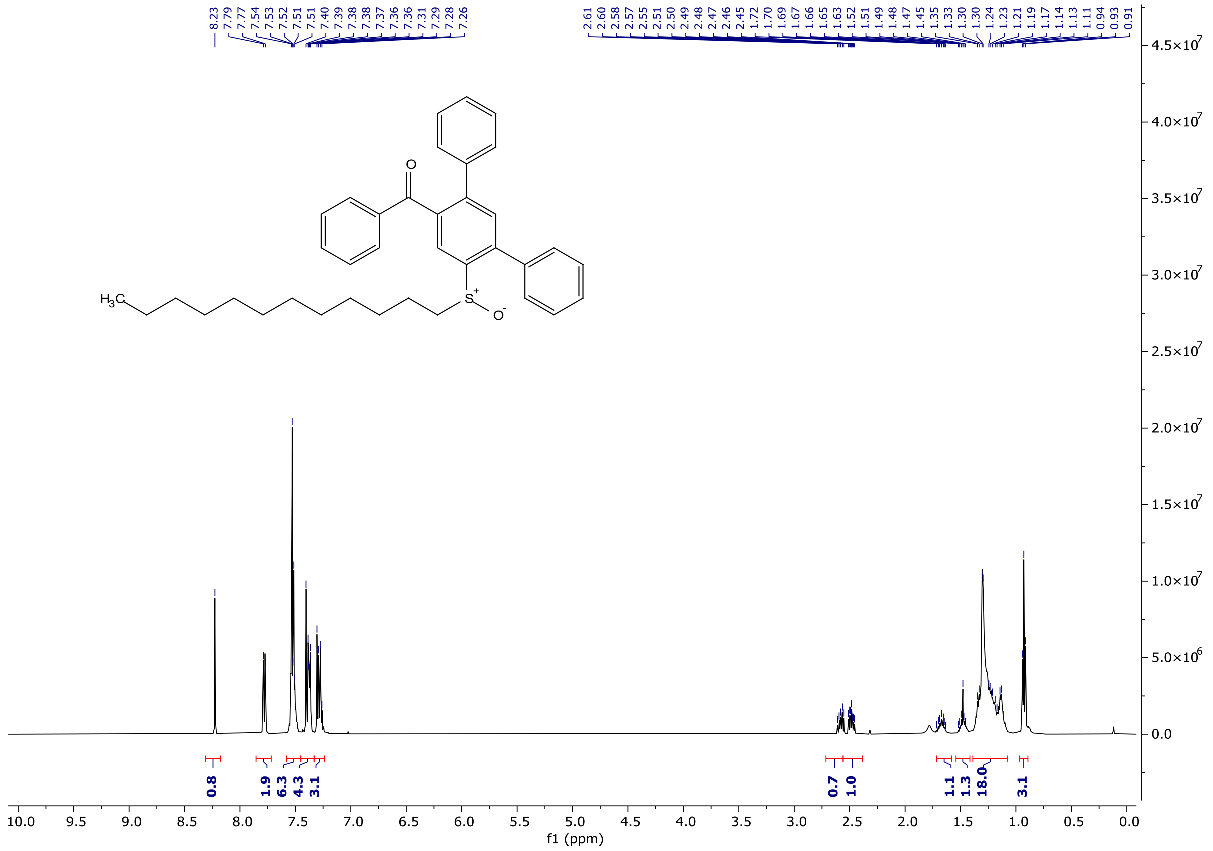

Figure S34. ^{1}H NMR (500 MHz, Chloroform-*d*) of compound **10p**.

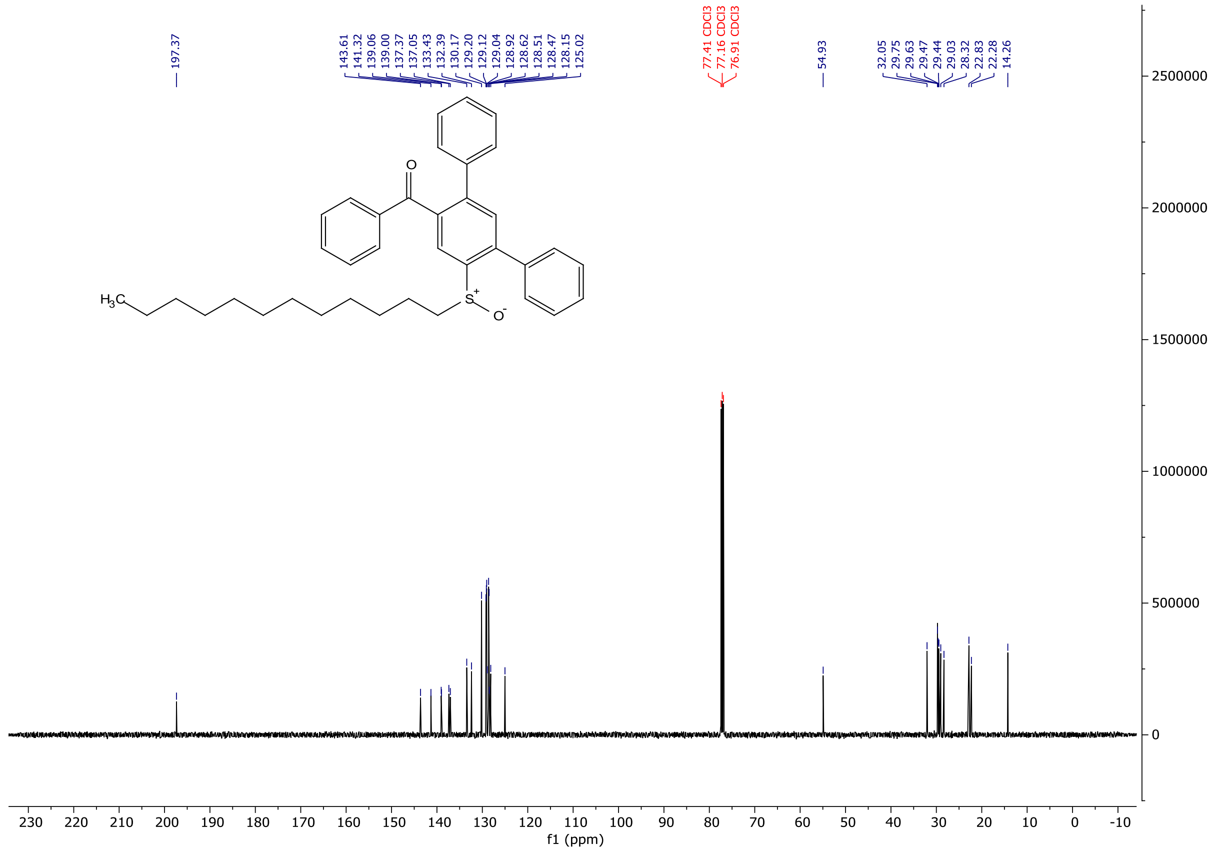

Figure S35. ^{13}C NMR (126 MHz, Chloroform-*d*) of compound **10p**.

Crystal structure data of compound 10a

Table S1: Crystallographic data

Molecular formula	$C_{32}H_{24}O_2S$
Space group	*P* $2_12_12_1$ (Nr. 19)
Crystal system	Orthorhombisch
Number of formula units, Z	4
Temperature/K	213,6(2)
a/pm	949,9(2)
b/pm	1464,9(3)
c/pm	1739,5(4)
V/10⁶pm³	2420,6(8)
Wave length/pm	71,073
Diffractometer	IPDS II, Fa. Stoe & Cie GmbH, Darmstadt
Number of measured reflexes	35706
Number of independent reflexes	6482
Number of refined parameters	319
$\delta_{min}/\delta_{max}$ (e/10^6pm^3)	-0,20/0,218
Absolute structure, flack parameter	1,09(10)
R_1 ($F_o > 4\ \sigma$)	0,0586
R_1 (all reflexes)	0,2697
wR_2	0,0728
GoF	0,753

Table S2: Position parameters and isotropic thermal deflection parameters. The standard deviations (in parentheses) refer to the last decimal place.

Atom	*x/a*	*y/b*	*z/c*	U_{iso}
S1	0,2774(2)	0,43277(7)	0,91833(8)	0,0464(4)
O2	0,4311(3)	0,4167(2)	0,9082(2)	0,060(1)
O3	0,4983(3)	0,7705(2)	0,0200(2)	0,064(1)
C1	0,1306(5)	0,5942(2)	0,0918(2)	0,034(1)
H1	0,0506	0,6006	0,1222	0,041
C4	0,2236(5)	0,5062(3)	0,8412(3)	0,038(1)
C5	0,2579(5)	0,5145(2)	0,9947(2)	0,032(1)
C6	0,2123(5)	0,8220(3)	0,1263(2)	0,039(1)
H6	0,2266	0,8328	0,0741	0,048
C7	0,4859(5)	0,7072(3)	0,0649(3)	0,040(1)
C8	0,2782(5)	0,5776(3)	0,7227(3)	0,050(2)
H8	0,3434	0,5909	0,6844	0,060
C9	0,2408(5)	0,6550(2)	0,1012(2)	0,029(1)
C10	0,7197(5)	0,7376(3)	0,1206(3)	0,046(2)
H10	0,7345	0,7795	0,0812	0,055
C11	0,1450(6)	0,6086(3)	0,7154(3)	0,043(1)
C12	0,2222(5)	0,7336(3)	0,1548(3)	0,035(1)
C13	0,0093(5)	0,4653(3)	0,0284(2)	0,033(1)
C14	0,1362(5)	0,5237(3)	0,0381(2)	0,033(1)
C15	0,3198(5)	0,5269(3)	0,7850(3)	0,044(2)
H15	0,4123	0,5068	0,7891	0,053
C16	0,8232(5)	0,7238(3)	0,1751(3)	0,0510(2)

H16	0,9078	0,7556	0,1720	0,061
C17	0,5951(5)	0,6901(3)	0,1239(3)	0,035(1)
C18	0,3630(5)	0,6434(3)	0,0591(2)	0,033(1)
C19	0,0181(5)	0,3703(3)	0,0264(2)	0,039(1)
H19	0,1049	0,3418	0,0323	0,047
C20	-0,1021(5)	0,3185(3)	0,0158(3)	0,049(2)
H20	-0,0956	0,2551	0,0144	0,058
C21	-0.1208(5)	0,05046(3)	0,0220(2)	0,043(1)
H21	-0,1288	0,5677	0,0253	0,052
C22	0,2015(5)	0,7192(3)	0,2319(3)	0,047(2)
H22	0,2054	0,6603	0,2517	0,056
C23	0,8007(5)	0,6631(3)	0,2339(3)	0,060(2)
H23	0,8692	0,6549	0,2716	0,072
C24	0,0480(5)	0,5900(3)	0,7729(3)	0,054(2)
H24	-0,0434	0,6124	0,7697	0,064
C25	0,1745(5)	0,7929(3)	0,2809(3)	0,057(2)
H25	0,1643	0,7829	0,3334	0,069
C26	0,1814(5)	0,8937(3)	0,1745(3)	0,055(2)
H26	0,1729	0,9524	0,1547	0,066
C27	0,0891(5)	0,5383(3)	0,8345(3)	0,048(2)
H27	0,0238	0,5246	0,8727	0,058
C28	-0,2402(5)	0,4527(3)	0,0108(3)	0,058(2)
H28	-0.3273	0,4810	0,0056	0,070
C29	0,6766(6)	0,6141(3)	0,2374(3)	0,065(2)
H29	0,6620	0,5721	0,2767	0,077
C30	0,3694(4)	0,5739(3)	0,0054(2)	0,035(1)
H30	0,4503	0,5669	0,9759	0,042
C31	0,5743(5)	0,6280(3)	0,1821(2)	0,048(2)
H31	0,4907	0,5951	0,1843	0,058
C32	0,0969(5)	0,6654(3)	0,6474(3)	0,075(2)
H32A	0,1448	0,6453	0,6018	0,112
H32B	0,1183	0,7285	0,6567	0,112
H32C	-0,0028	0,6583	0,6406	0,112
C33	-0,2303(6)	0,3598(3)	0,0074(3)	0,059(2)
H33	-0,3106	0,3246	0,9994	0,071
C34	0,1629(5)	0,8789(3)	0,2525(3)	0,054(2)
H34	0,1428	0,9275	0,2851	0,065
